# Large-scale labeling and assessment of sex bias in publicly available expression data

**DOI:** 10.1186/s12859-021-04070-2

**Published:** 2021-03-30

**Authors:** Emily Flynn, Annie Chang, Russ B. Altman

**Affiliations:** 1 Biomedical Informatics Training Program, Stanford University, Stanford, CA USA; 2 Program in Human Biology, Stanford University, Stanford, CA USA; 3 Department of Bioengineering, Stanford University, Stanford, CA USA; 4 Department of Genetics, Stanford University, Stanford, CA USA; 5 Department of Medicine, Stanford University, Stanford, CA USA

## Abstract

**Background:**

Women are at more than 1.5-fold higher risk for clinically relevant adverse drug events. While this higher prevalence is partially due to gender-related effects, biological sex differences likely also impact drug response. Publicly available gene expression databases provide a unique opportunity for examining drug response at a cellular level. However, missingness and heterogeneity of metadata prevent large-scale identification of drug exposure studies and limit assessments of sex bias. To address this, we trained organism-specific models to infer sample sex from gene expression data, and used entity normalization to map metadata cell line and drug mentions to existing ontologies. Using this method, we inferred sex labels for 450,371 human and 245,107 mouse microarray and RNA-seq samples from refine.bio.

**Results:**

Overall, we find slight female bias (52.1%) in human samples and (62.5%) male bias in mouse samples; this corresponds to a majority of mixed sex studies in humans and single sex studies in mice, split between female-only and male-only (25.8% vs. 18.9% in human and 21.6% vs. 31.1% in mouse, respectively). In drug studies, we find limited evidence for sex-sampling bias overall; however, specific categories of drugs, including human cancer and mouse nervous system drugs, are enriched in female-only and male-only studies, respectively. We leverage our expression-based sex labels to further examine the complexity of cell line sex and assess the frequency of metadata sex label misannotations (2–5%).

**Conclusions:**

Our results demonstrate limited overall sex bias, while highlighting high bias in specific subfields and underscoring the importance of including sex labels to better understand the underlying biology. We make our inferred and normalized labels, along with flags for misannotated samples, publicly available to catalyze the routine use of sex as a study variable in future analyses.

**Supplementary Information:**

The online version contains supplementary material available at 10.1186/s12859-021-04070-2.

## Background

Sex differences have been reported across multiple traits and diseases and in response to drugs. In the case of drug response, women experience more than 1.5-fold as many adverse drug events [1]. This is in part due to historical exclusion of women from clinical research. In 1993, the policies excluding women were revoked and the National Institutes of Health (NIH) Revitalization Act was passed to increase inclusion of women and minorities in clinical research. This has improved inclusion of women, but clinical studies continue to show sex bias against female participants [2–4]. Additionally, preclinical studies are critical to the drug development process [5]; however, there is limited reporting of sex in both rodent [6, 7] and cell line research [8]. In 2016, the NIH passed a mandate that requires researchers to consider sex as a variable in preclinical analysis [9], which led to increases in sex reporting, but sex bias in these studies still remains [10].

Gene expression data is often used as part of the drug development pipeline in order to better understand cellular and molecular-level effects of drugs and assess their mechanisms of action and side effects [11]. While we do not expect all drugs to show cellular-level sex differences in drug response, pervasive use of single-sex studies may lead to the development of drugs that do not work well for both men and women.

Although multiple studies have sought to assess sex reporting and bias in specific areas, including in skin [12], neuroscience [6, 13], and pain research [14], and across biomedical fields [6, 10], these assessments largely focus on scientific literature. Public repositories of biological data provide another avenue for assessing sex bias. Repositories such as Gene Expression Omnibus (GEO) [15], Sequence Read Archive (SRA) [16], and ArrayExpress [17] contain gene expression data and corresponding metadata describing the experiments and samples, allowing for re-analysis and re-use of these data. Gene expression metadata is organized according to the Minimum Information about a Microarray Experiment (MIAME) [18] and Minimum Information about a high-throughput Nucleotide Sequencing Experiment (MINISEQE) guidelines, despite this, metadata is heterogeneous across experiments, making large-scale analysis difficult. In the case of examining sex labels, not only are these labels reported inconsistently, the majority of samples are missing this information, limiting assessment of sex bias. Previous studies also suggest there may also be widespread misannotation [19, 20]. Metadata normalization, such as that performed by MetaSRA [21], seeks to address the problem of inconsistent reporting of sex, cell type, age, and multiple other labels by mapping these to existing ontologies; but is unable to address missing metadata.

Assessment of sex bias in gene expression data does not require metadata: sex can be imputed from the expression levels of X and Y chromosome genes. Recount2 [22] used expression data to impute sex labels across 70,000 publicly available human RNA-seq samples using a linear model and found slight female bias overall [23]; however, their work does not extend to microarray platforms [23]. While other methods for imputing sex labels from microarray expression data have good performance [20, 24], they are either clustering based and therefore limited to mixed sex studies (because they assume two clusters) [20, 24] or only work for specific platforms [25].

In addition to challenges with metadata, expression data can be difficult to study at scale because of platform heterogeneity and differences in data pre-processing and normalization [26]. Previous resources, such as ARCHS4 [27] and recount2 [22], have worked to address these limitations by releasing human and mouse RNA-seq data that has been processed with standardized pipelines.

Refine.bio is a new transcriptomic resource that addresses many of these previous limitations (http://www.refine.bio, [28]). It contains all publicly available microarray and RNA-seq data from more than twenty-two organisms, pre-processed, de-duplicated, and normalized to allow for examination across platforms. Additionally, portions of the metadata are “harmonized”, meaning that groups of semantically similar labels (e.g., “tissue” and “organ”) have been manually aggregated into categories.

We sought to assess sex bias at the sample and study level across all publicly available expression data, including specifically drug-related datasets. For this analysis, we focused on human and mouse expression studies, using refine.bio as a resource, and expanded on previous analyses by using a penalized logistic regression model to predict sample sex. In the process, we also considered cell line sex and used our imputed sex labels to estimate baseline misannotation rates.

## Results

### Aggregation of publicly available mouse and human expression data

**Fig. 1:**
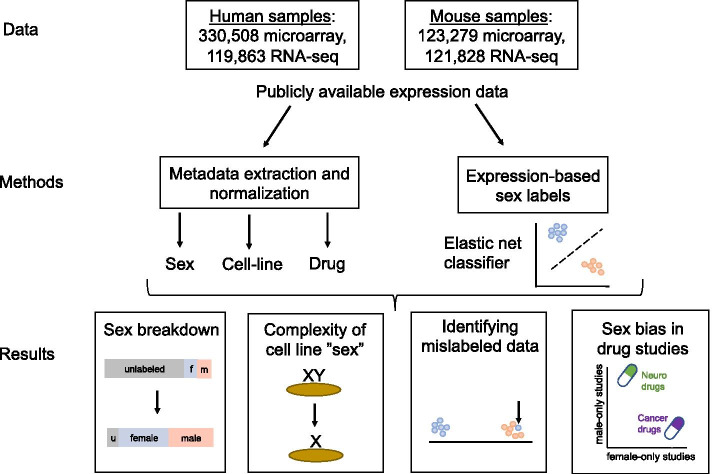
Schematic of study analysis. We constructed our dataset from all human and mouse microarray and RNA-seq data in refine-bio. We then extracted metadata labels for sex, cell line, and drug, and built elastic net models to infer sample sex from gene expression. Using the metadata and inferred sex labels, we investigated the sex breakdown of samples by organism, as well as cell line “sex” complexity (e.g., Y chromosome loss). We also looked for the presence of mislabeled data by examining concordance between predicted and actual sex labels, and at sex bias in drug studies (e.g., underrepresentation of females in studies of nervous system drugs)

We downloaded all expression data and metadata for all human and mouse samples and studies from refine-bio. After filtering (see “Expression data pre-processing” section), this resulted in 330,508 and 123,279 microarray samples (spanning 11,333 and 9303 studies) and 119,863 and 121,828 RNA-seq samples (spanning 6240 and 6477 studies) in human and mouse respectively (Fig. 1).

### Large-scale sex labeling of mouse and human data

#### The majority of samples are missing metadata sex labels

**Fig. 2:**
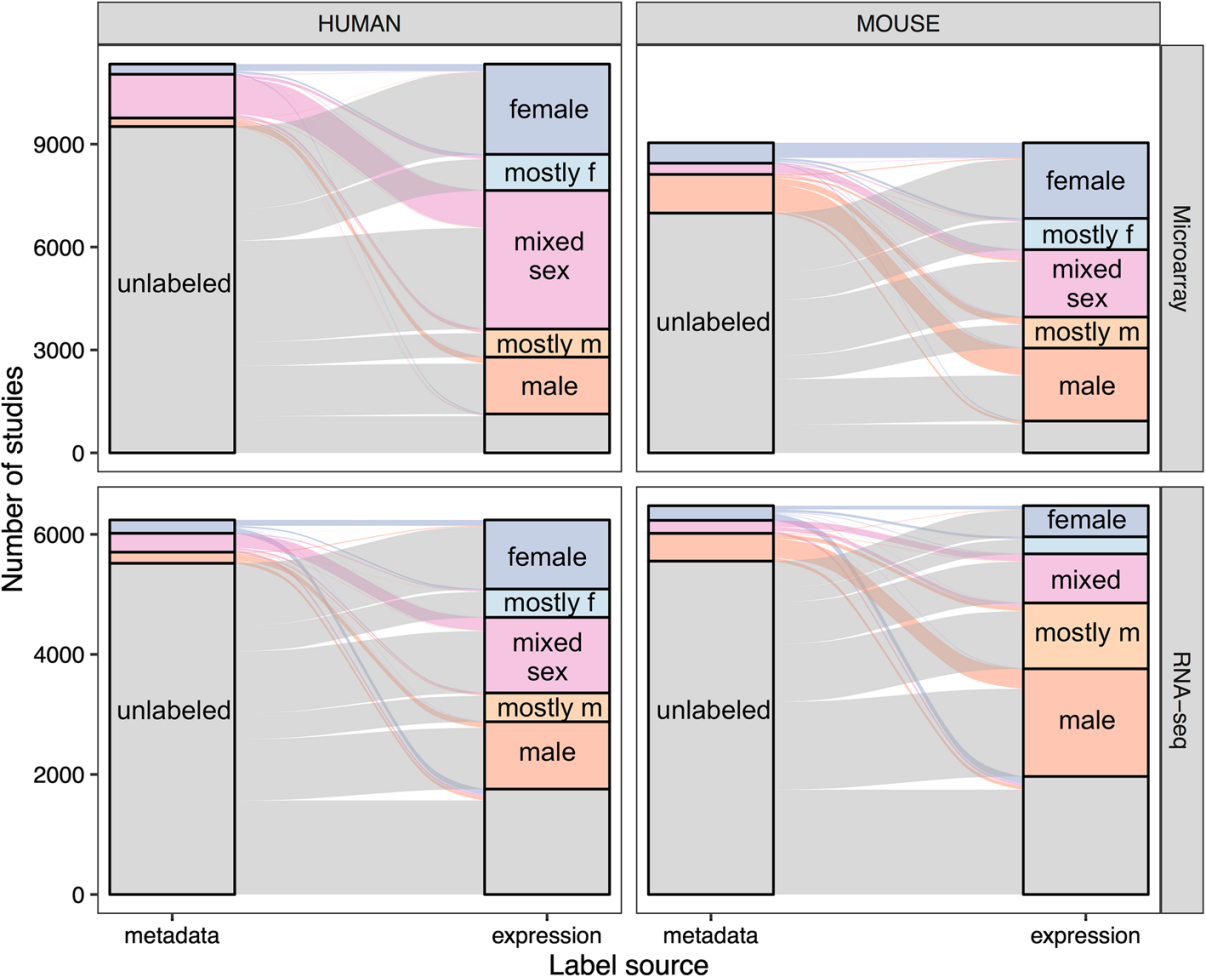
Breakdown of study sex labels. Alluvial diagram showing the breakdown of study sex labels in the metadata (left of each panel) and after expression based labeling (right of each panel). The flow is colored by the initial metadata labels and helps trace whether there is a “change” in labels. For the majority of studies with metadata labels, the labels match the inferred expression labels. The results are shown for both human and mouse (columns) and in microarray and RNA-seq (rows). Gray indicates that a study is missing sex labels (for studies with up to 60 samples, more than half of the labels are missing: for studies with greater than 60 samples, there are fewer than 30 labels), dark blue means the samples in the study are female-only, dark orange is male-only, and pink is mixed-sex. Mostly male (light orange) and mostly female (light blue) indicate that more than 80% of the samples labeled in that study are of that sex. For the metadata breakdown, the numbers of studies in mostly-male and mostly-female categories was small (e.g. 71 mostly-female and 87 mostly-male studies in human microarray) and were grouped into mixed sex for ease of visualization. (For a similar figure with sample sex breakdown see Additional file 1: Fig. S1.)

We extracted and examined the missing of metadata sex labels in all mouse and human data present in refine.bio (see “Metadata sex label extraction” section). Across all four datasets (human, mouse, microarray and RNA-seq), we find that the majority of samples and studies do not have metadata sex labels (left portion of each panel in Fig. 2, Additional file 1: Fig. S1). For example, in human microarray, we find that 70.7% of samples and 83.9% of their corresponding studies are missing sex labels (see Additional file 2: Table S1A and B for RNA-seq and mouse statistics). In the absence of metadata sex labels, we can neither assess inclusion of males and females in studies nor examine whether there are sex-related effects*.*

#### Inferring sample sex from expression data

Existing methods for inferring missing sex labels from expression data are limited to mixed sex studies [20, 24], so cannot be applied across studies where we do not know the sex breakdown (e.g. some studies could be single-sex), or are only applicable to certain platforms or data types [23, 25]. In order to overcome these limitations and label the majority of publicly available human and mouse expression data, we trained multiple machine learning models to impute sample sex from the expression of X and Y chromosome genes. We selected penalized logistic regression because of its high performance and interpretability (see “Model selection and training” section for a description of other models used and Additional file 1: Fig. S2 and Additional file 2: Table S2 for the results of using these models). The predicted value from the model corresponds to the model’s predicted probability of that sample being male (P(male) or P(sex = 1) using the standard coding where female is 0 and male is 1); we refer to this value as a *sample sex score*. We both use this score to label sample sex, assigning samples to male or female at a certain threshold cutoff, and examine the distribution of these scores across samples.

We assessed the accuracy of our models in a held-out test set (agreement 91.7% in microarray, 88.4% in RNA-seq for human) and as compared to all metadata sex labels (93.1% in microarray, 91.3% in RNA-seq for human) (see Additional file 2: Table S3A for test set composition). The flow of sample labels highlighted in Fig. 2 shows the correspondence between metadata and inferred expression labels. We see similar performance in human and mouse (see Additional file 2: Table S3B), with higher agreement in microarray datasets than RNA-seq. We additionally looked at the performance for the subsets of these labels in single sex studies (95.1%), mixed sex studies (91.5%) (statistics shown for human microarray), and, in human, manually annotated sex labels from a previous analysis (94.2%) [25]. As expected, at more stringent cutoffs for assigning sample sex, we achieved higher concordance at the expense of leaving a portion of samples unlabeled (Additional file 1: Fig. S3, values in Additional file 2: Table S3B). We selected a threshold of 0.7 to correspond with approximately 95% agreement between metadata and expression labels across the datasets. Our models show good performance in most platforms; however, 7 of 62 platforms (covering < 3% of all samples) have very poor performance in the extended test set (agreement < 70%, see Additional file 1: Fig. S4 and Additional file 2: Table S4A for platform-specific accuracy). We filtered to remove these “problem platforms”, and at a threshold of 0.7, for human data, we have 94.3% and 92.1% concordance with 92.9% and 70.0% of the data labeled in microarray and RNA-seq respectively (mouse datasets show similar patterns, Additional file 2: Table S4B). RNA-seq samples with more read counts are more likely to be labeled (point biserial correlation = 0.197, *p* < 2 × 10^–16^); higher read counts are also correlated with increased accuracy of labeling, but to a lesser extent (point biserial correlation = 0.0251, *p* < 4.55 × 10^–5^, Additional file 1: Fig. S5).

#### Key model features include Y chromosome and X escape genes

Elastic net performs feature selection, reducing the number of genes (or transcripts) included in the model. We examined the location and X-inactivation status of the resulting genes (see Additional file 1: Fig. S6 and Additional file 2: Table S5). For human microarray, this reduced the number of genes from 548 to 33, none of which were located in the pseudoautosomal region. The genes with the strongest coefficients in the male direction are located on the Y chromosome, while the genes with the strongest coefficients in the female direction are known X chromosome escape genes [29]. This finding is consistent with biological understanding; X chromosome genes that escape inactivation have higher expression in females because there are two X chromosomes. A small number of inactive and variable escape X chromosome genes are included in the model with small coefficients in both directions.

#### Sex labeling mixed sex or pooled samples

A small fraction of samples (< 0.01% in human data, 0.6% in mouse data) have metadata indicating that they are pooled or mixed sex samples. This practice is more common in mouse data, as samples are often pooled from mice of the same strain before analysis, and it is used to both increase signal and reduce the number of expression samples (and thereby the cost). Pooling often includes samples from both sexes, but this is highly variable. We sought to examine our models’ predictions on these mixed or pooled samples. The models’ sample sex score distributions are significantly different in mixed/pooled samples versus male or female labeled samples (see Additional file 1: Fig. S7). At a threshold of 0.7, which was selected to correspond to a 95% accuracy rate (Additional file 1: Fig. S4), pooled samples fall into the unlabeled category at higher rates than samples with male and female metadata sex (in mouse microarray data, 28.8% of pooled versus 5.4 and 7.7% of female and male samples are not labeled).

#### Sex breakdown shows slight female bias in humans and male bias in mice

We applied these models to all publicly available human and mouse expression data and found that the overall sex breakdown is slightly female-biased (52.1%) in humans and male-biased (62.5%) in mice (Additional file 1: Fig. S1; for RNA-seq versus microarray breakdown see Additional file 2: Table S1A). At a study-level, in humans, the majority of studies are mixed sex and there are more female-only than male-only studies (55.3% mixed sex, 25.8% female-only, 18.9% male-only). In mice, single sex studies make up the largest proportion of studies; however, this is only slightly more than the fraction of mixed sex studies, and there are more male-only than female-only studies (47.4% mixed sex, 21.6% female-only, 31.1% male-only) (right portion of each panel in Fig. 2). This pattern does not appear to change over time (Additional file 1: Fig. S8).

### Cell line “sex” is complex

We performed named entity recognition to identify cell lines based on the metadata and mapped 74,140 out of 99,426 human samples and 8433 out of 17,061 mouse samples to Cellosaurus identifiers. For human RNA-seq samples, this showed high concordance with MetaSRA (76.5% exact matching, 14,390 out of 18,821 samples).

#### Our analysis supports previous observations that cell line “sex” is fundamentally different from tissue sex

**Fig. 3:**
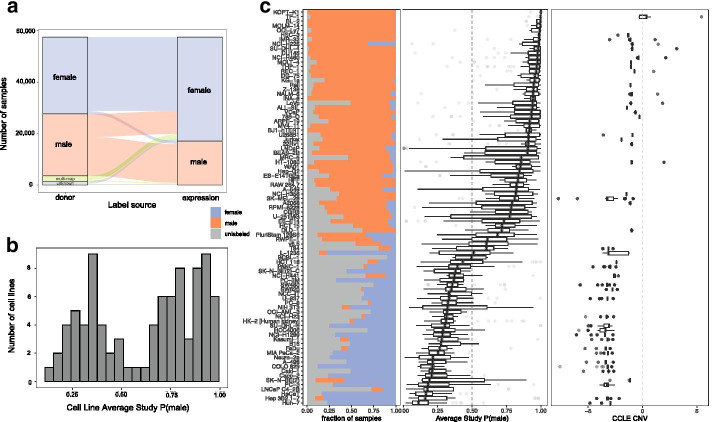
Y chromosome loss is prevalent and variable across cell lines. **a** Cell line sex label switching. The sex of the donor cell line is on the left and the imputed sex is on the right. Samples are divided into female (blue) and male (orange). Additionally for donor cell sex labels, we include samples with unknown sex (gray) and samples with metadata mapping to more than one cell line (green). **b** Distribution of average study sex scores (e.g. P(male) for a sample) for male cell lines shows a bimodal pattern, indicating that many of these cell lines appear “female-like” (see Additional file 2: Table S7 for a list). **c** Profiles of Y chromosome loss across male cell lines with more than five studies. Each cell line is a row. From left to right, the first panel shows the fraction of male (orange), female (blue), and unlabeled (gray) samples, the second shows the distribution of study average sample sex scores, and the third shows the cell line specific distributions of Y chromosome gene copy number (CNV) from the Cancer Cell Line Encyclopedia [31]. Overall, this demonstrates the highly variable and cell line-specific nature of Y chromosome loss

While previous studies [8] have encouraged researchers to report the sex of their cell lines, other studies have shown that cell line sex can be complicated, in part because cell lines often lose their Y chromosomes in culture [30]. We find that many samples with reference sex male are labeled as female based on expression data (37.8%) while relatively few samples have reference sex female and are labeled male (4.24%, Fig. 3A, Additional file 1: Fig. S9). This pattern of cell line “switching” from male to female matches patterns that have already been shown on a smaller scale; namely, many cell lines lose Y chromosomes.

Overall, we see significant enrichment of the overall proportion of female imputed labels in cell line versus tissue samples (Additional file 1: Fig. S10A, *p* < 0.05 for human and mouse microarray and human RNA-seq, N.S. for mouse RNA-seq). Additionally, the distributions of sample sex scores for cell line versus tissue show increased female scores and a wider distribution of scores in cell line versus tissue data (Additional file 1: Fig. S10B).

#### Our analysis provides evidence of cell-line specific patterns of Y chromosome loss.

Across male cell lines, the fraction of inferred male versus inferred female samples varies greatly, with certain cell lines appearing more “male-like” (e.g., THP-1, OCI-Ly7), “female-like” (e.g. KYSE-30, HaCaT), and other cell lines appearing to belong more in the middle with samples belonging to both (A549, HCT-116) (Fig. 3b, c). We further examined 87 cell lines from male donors that had a large number of studies (≥5) and samples (≥3 per study) (Additional file 2: Table S6). Of these cell lines, 45 are included in Cancer Cell Line Encyclopedia (CCLE) [31]. On a cell line level, our sample sex score predictions correlate with CCLE Y but not X chromosome copy number (Spearman correlation of medians for Y: 0.774, *p* < 4.58 × 10^–10^, and X: − 0.0944, *p* = 0.537) (Fig. 3C).

### Our models allow for improved detection of mis-annotated data

We can estimate metadata mis-annotation rates at scale by comparing our inferred sex labels to metadata sex labels. First, we examined the mismatch rates in mixed sex versus single sex tissue studies and found significantly higher mismatch rates in samples from human mixed sex studies (4.87%) than single sex studies (2.03%) (Chi-squared *p *value < 1.69 × 10^−48^). By contrast, in mouse studies, mismatch rates for samples in single sex studies (3.42%) exceeded that of mixed sex (2.58%) studies (*p* < 9.70 × 10^−6^) (Additional file 2: Table S7A).

Second, we created a dataset of large mixed sex studies with metadata labels, consisting of 6066 mouse and 8658 human samples (168 and 163 studies respectively), which we sex labeled with the Toker and massiR methods, as well as our own (see “Comparing expression-based methods in mixed sex studies” section). Across these samples, 4.74% of human and 6.64% of mouse samples had metadata sex labels that did not match any of the predicted expression-based labels. At a study level, 35.0% of human and 16.7% of mouse studies contained at least one mismatched sample (Additional file 2: Table S7B).

Third, to estimate the probability that an individual sample is mislabeled in a mixed sex study, we fit a mixture of Gaussians to each study-level distribution of sample sex scores (see “Clustering to identify high confidence swaps in mixed sex studies” section). This allowed us to identify high confidence mismatches at a particular probability threshold (> 0.95) by examining the concordance between metadata labels and expression labels. After discarding mixed sex studies where the best model was a single Gaussian (409 or 26.9% of mixed sex studies—by comparison, we also clustered single sex studies and found 94.0% of single sex studies were best modeled by a single Gaussian), we estimate that 2.04% of mouse and 3.06% of human samples are mislabeled in these studies. At a study level, this corresponds to 26.0% of mouse and 51.7% of human studies containing at least one high confidence mismatched sample (Additional file 2: Table S7C).

### Sex breakdown of drugs in tissue data

#### Labeling drug studies using metadata

We identified all gene expression studies containing drug mentions by applying named entity recognition and normalization to the study and sample metadata (see “Drug labeling” section). We labeled studies with *drug mentions* (n = 7665), which contain a drug in the study abstract or title, and *drug exposure* studies (n = 1095) which include a drug in the sample treatment field.

Out of the 95,788 human and mouse samples with non-null treatment fields (spanning 9130 unique treatment labels), 30,000 mapped to only control terms (1144 unique) and 463 mapped to both DrugBank drugs and control terms (91 unique), 12,692 mapped to only drugs (1410 unique treatment labels, mapping to 417 drugs), leaving 52,633 unmapped treatment labels (6485 unique). 96 additional samples mapped using compound or title.

The overlap between the studies with drug mentions (n = 7665) and treatment studies (n = 1095) is 757 studies; 557 of these studies map to the same drug or drug(s) and 159 map to at least one of the same drugs. After filtering for only overlapping drugs, there are 844 unique study-drug pairs spanning 319 drugs and 715 studies. We include a list of these *drug exposure studies* in Additional file 2: Table S8A.

#### The sex breakdown of drug studies shows limited sex bias overall

**Fig. 4:**
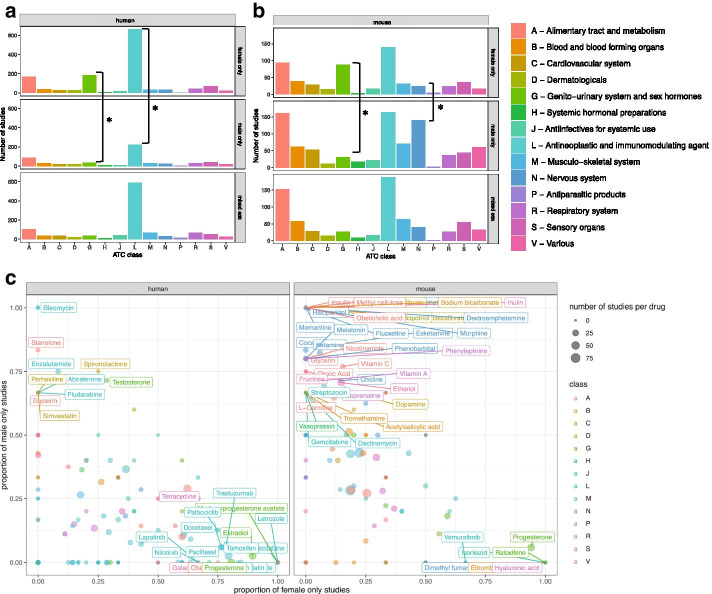
Sex breakdown by drug ATC class in human and mouse data. The breakdown shows enrichment of female only studies in human cancer drugs and male only studies in mouse drugs. Count breakdown of female only, male only, and mixed sex studies by class in humans (**a**) and mice (**b**) (* indicates *p* < 0.05 after Bonferroni correction). The fraction of studies for each individual drug is also shown in (**c**), where the x axis is the fraction of female only studies and the y axis is the fraction of male only studies. Each point is a drug, the size of the point indicates the number of studies that include that drug, and the color of the point is ATC class. Drug name labels are included for drugs with at least three studies and a strongly sex-biased ratio ($$> \,{\raise0.7ex\hbox{$2$} \!\mathord{\left/ {\vphantom {2 3}}\right.\kern-\nulldelimiterspace} \!\lower0.7ex\hbox{$3$}}$$ of studies only one sex, $$< \,{\raise0.7ex\hbox{$1$} \!\mathord{\left/ {\vphantom {1 3}}\right.\kern-\nulldelimiterspace} \!\lower0.7ex\hbox{$3$}}$$ the other); other labels are omitted for ease of visualization. For a full list of drugs see Additional file 2: Table S8

We then asked the question of whether global patterns of sex breakdown continue in the context of drug studies. We found that this was the case, with slightly increased female-bias in human studies (52.1% overall, 59.9% in drug studies) and decreased male-bias in mouse studies (62.5% overall, 57.1% of drug studies) (Additional file 1: Fig. S11). This pattern varies across drug Anatomic Therapeutic Class (ATC), with female-only studies enriched in human and mouse genitourinary system drugs (class G, *p* < 10^–8^ and *p* < 10^–16^ respectively) and in human cancer/immune system drugs (L, *p* < 10^–7^), and male-only studies were enriched in mouse nervous system drugs (N, *p* < 10^–7^) (Fig. 4). These patterns match sex breakdowns in drug exposure studies (Additional file 1: Fig. S2A, C) and a previous dataset of manually curated drug expression studies ([32], n = 472) (Additional file 1: Fig. S12B, D).

#### Human cancer drug studies are female-biased

There are 370 female-only versus 105 male-only studies with cancer drug mentions; however, the majority of cancer drug studies are still mixed sex (n = 396). These studies span 143 different drugs and despite being highly female-biased at the study level, 115 of these drugs (80.4%) have at least one mixed sex study.

To determine the possible association between cancer and sex, we examined whether drugs targeting strongly sex-biased cancers (e.g. breast, ovarian, prostate) were associated with more single sex studies. In humans, of the drugs with > 70% female-only studies, 9 out of 21 are breast cancer specific and 19 out of 21 drugs contain indications for breast or ovarian cancer (Additional file 2: Table S9A). For drugs with > 70% male only studies, in humans 2 out of 8 of these are used for prostate cancer (Enzalutamide, Abiraterone) and the remainder are used for multiple types of cancer (this includes Bleomycin which is used for multiple cancers including ovarian).

#### Mouse nervous system drug studies are male-biased

In the case of mouse nervous system drugs, we see high male-bias in the proportion of male mice used as study subjects. This pattern of male-only rodent studies in neuroscience has been previously reported [6]. At an individual drug level, while the majority drugs have both single and mixed sex studies (n = 147, 38.7% of human; n = 95, 34.5% of mouse) or mixed sex only studies (n = 115, 30.3% of human; n = 38, 13.8% of mouse), some drugs had only male studies (10.3% of human, 20.7% of mouse). In nervous system drugs in particular (n = 27 in humans, n = 37 in mice), this fraction is markedly increased in mouse nervous system drugs, with 13 drugs that are only studied in males versus 5 only in females (see Additional file 2: Table S9B for a list of neuro drugs). The 13 drugs with male-only studies in mice, two have mixed sex studies in humans (Heroin and Carbamazepine), two only have male-only studies in humans (Fluoxetine and Haloperidol), and the remainder have no human studies (Acepromazine, Amphetamine, Levadopa, Memantine, Modafinil, Olanzapine, Quetiapine, Salsalate, Venlafaxine). Fluoxetine is particularly interesting because it is a commonly prescribed antidepressant and is present in eight mouse studies, all of which are male-only. The eight studies are all treatment (fluoxetine) versus control comparisons and collectively contain 128 samples all from mouse brain tissue. In humans, there is only one fluoxetine study and it is also all male.

## Discussion

We inferred sample sex labels at scale from expression data; to our knowledge, our analysis represents the first effort to label the majority of publicly available human and mouse microarray and RNA-seq expression samples. We leveraged these labels to assess sex bias overall and in drug studies, examine cell line sex, and estimate misannotation rates. Below, we discuss our findings in the context of previous results, and potential limitations of our methodology.

### Labeling expression data at scale

Improving on previous methods, which have high accuracy but are focused on specific data or platforms [23, 25] or mixed sex studies [20, 24], our labeling method has consistent accuracy across the majority of platforms and in both mixed and single sex studies. We leverage the sample and study metadata to label sample sources (e.g. tissue, cell line, primary cell, etc.) and map samples and studies to cell line and drug identifiers from Cellosaurus [33] and DrugBank [34] respectively. Our combined metadata mappings and inferred sex labels allow us to examine the sex breakdown across sample source type, cell lines, and drug-related expression studies.

We found that the overall sex breakdown shows slight female-bias in human samples and male-bias in mouse samples. This breakdown continues at a study level, where about half of studies are mixed sex but there is still slight female- and male-bias in human and mouse studies respectively. This low overall sex bias is in contrast to what we expected, given the history of clinical trials excluding women, but is consistent with previous findings in human RNA-seq data [23]. In preclinical rodent research, male-bias often comes from the historical (and incorrect) perception that female rodents show more variability due to hormonal cycles [35, 36], and in our analysis, we also see male-bias but to a limited degree. It is likely that analysis by disease or sub-field may show different patterns of sex bias. It is important to note our analysis focuses on publicly available expression studies and does not extend to private data from drug company data or clinical trials.

When we examined labels over a fifteen-year period (2004–2019), we found that the missingness of sex labels and patterns of sex bias did not appear to change over time. This was also unexpected because in 2016 there was an NIH mandate to include sex as a variable in preclinical studies and overall there has been increased awareness of its importance. Specific fields have documented improvements in the inclusion of females and reporting of sex [10]; it is possible that future analysis grouping by field may show changes in the sex breakdown over time despite the overall pattern seeming consistent.

### Sex labeling cell lines shows Y chromosome loss

In 2014, Shah et al*.* [8] encouraged researchers to include and examine the sex of the cells they use. Previous studies have shown that certain types of male and female cells in culture respond differently to drugs [37], underscoring the importance of this consideration. However, there is an important distinction between examining the sex of primary or stem cells and examining the sex of transformed cell lines. While the sex of the cell line donor provides some information, cell lines in culture often undergo chromosomal loss or duplication [38], in particular, loss of Y chromosomes is common [30]

While the gold standard for cell line sex labeling is PCR-based authentication [39], in the absence of this information, we can use our models to infer sample sex and identify Y chromosome loss from expression data, allowing for re-examination of existing studies. We found both highly prevalent (43.4% of male cell lines contain at least one female-appearing sample) and variable patterns of Y chromosome loss across cell lines. It is unclear whether this variability across cell lines is due to differences in cell stability [40], passage number (Y chromosome loss is more common after longer time in vitro [38]), contamination, or mislabeling. We additionally found that the distribution of sample sex scores in cell lines varies greatly from that of tissues and primary cells, with human primary cell line distributions more closely resembling tissues than cell lines. Many mouse primary samples had ambiguous sample sex scores, which is likely due to the practice of using pooled samples for mouse primary cultures. Altogether, these results highlight the lack of a cell line sex binary and match previous recommendations against using cell lines for examining sex-related effects [41]. We performed the remainder of the analysis in cell lines and tissues separately.

While we infer potential Y chromosome loss in cell lines with donor sex male that appear female in our expression data, it is also possible that these cell lines are contaminated, which is very common [42]. Proper cell line authentication (via PCR) is necessary to confirm contamination; however, this is not possible during re-analysis of existing studies. Our estimates are limited in that they do not provide direct copy number information, which is important because cell lines also often undergo X chromosome duplication or changes in autosomal ploidy [38]. However, the sex breakdown of individual cell lines correlates with the cell line’s Y chromosome reference copy number variation (CNV) statistics from Cancer Cell Line Encyclopedia (CCLE), validating our inferred labels and providing further evidence of cell line specificity.

Another potential factor that may influence sex-related behavior in cell lines is the presence of media, which we did not examine in this analysis. Many media contain phenol red as an indicator, which has estrogenic properties, and serum, which mimics a pregnancy-like environment, and these may impact the study of sex-related effects [41, 43]. Examining the effects of media is very challenging as there is great heterogeneity and media types are often underreported or missing specific information [43].

### Estimation of misannotation rates

Metadata mislabeling is a widespread problem. Previous studies [19, 20] have used inferred expression labels to estimate mislabeling rates. Toker et al. found that 46% of the 70 mixed sex datasets they examined contained mislabeled samples, with an overall sample mislabeling rate of 2% (4160 samples). In humans, we estimate that 2% of samples contain misannotated sex labels and 52% of studies contain at least one misannotated sample. Our estimates match previous studies [19, 20] and expand this analysis to include many more microarray platforms, RNA-seq studies, and mouse samples.

We initially examined the difference in mismatch rates between mixed and single sex tissue studies, with the expectation that mixed sex studies would have higher mismatch rates than single sex studies because of the potential for swapped annotations. This was the case in human but not mouse data, and it is unclear why single sex studies show higher mismatch rates in mice. It is possible that our approach may have poorer performance in mouse data than human data at identifying cell line samples (for human data, we evaluated through comparison to MetaSRA). Inclusion of cell line samples within the tissue analysis would increase mislabeling rates*.* Additionally, this difference in rates may be related to the collection of samples from mouse pups, which are hard to sex [44]. This raises questions about the proportion of mouse studies that are truly single sex, which requires further investigation and underscores the importance of using proper authentication for sex determination.

### Including sex as a variable in drug-related studies

The sex breakdown of drug data shows limited sex bias and matches the overall breakdown of samples and studies; however, studies of specific drug classes show sex bias: particularly mouse nervous system drugs (male-biased) and human cancer drugs (female-biased). While we focused our analysis on studies that mention a drug, which may have low specificity, we found a similar sex breakdown in drug exposure studies, and in a crowd-sourced set of drug studies from CREEDS [32]. Future work may involve better annotation of drug exposure studies from metadata using deep learning based methods.

While it is of interest to examine whether particular drugs lead to differential gene expression responses between males and females, we are underpowered to identify interaction effects in most of these studies using classical methods because of sample size. However, it is possible that non-parametric methods and pathway or gene set based techniques could be used to examine these effects [45]. Additionally, it is still important to include both sexes in studies regardless of our ability to examine interaction effects [7]. Our inferred sex labels allow for assessment of these practices. In addition, we hope that these labels can lead to improved selection of studies and inclusion of sex as a variable in the re-analysis of public data.

### Analysis limitations

In developing our sex labeling model, we chose to focus on a model that would allow us to infer sample sex at high accuracy across a wide range of samples, studies, and platforms. Our model has better performance on microarray data, which may be due to the fact that we used similar models for microarray and RNA-seq data by applying the Box-Cox transformation to the RNA-seq count data. It is also possible that alternate models or transformation methods for count data [e.g. the voom transform [46]] may perform better. Additionally, the high degree of homology between X and Y chromosomes can lead to technical artifacts during RNA-seq read mapping that make discerning sample sex more difficult. While these artifacts are present, they are limited and we still achieve good labeling performance. Use of methods to directly address these artifacts, such as XYalign [47], may also improve performance. As is common, our model results in one probability score for each sample, allowing for accurate probability cutoffs. However, it is possible that having two predicted scores (for male-like and female-like behavior) could allow for better understanding of sex in cell lines, pooled samples, and samples from intersex individuals. While our analysis uses the expression of X and Y chromosome genes to label sample sex, we did not consider autosomal expression because previous studies have indicated that sex differences in this are generally tissue-specific [48]. However, sex differences in autosomal expression, as determined through methods such as ISEXs [49], are also important for biological understanding and future work may involve including autosomal genes.

As part of our analysis, we also leveraged metadata to map samples and studies to sample source types, cell lines, and drugs. For mappings of samples to cell line and drug labels, we required an exact match with a cell line name or synonym, and for sample source type, we also required exact lexical matches for many of the sample source categories, resulting in many unlabeled or “other” samples. Use of state-of-the-art biomedical named entity recognition and normalization methods may help to improve the accuracy and sensitivity of these labels.

We focused on sex bias in publicly available human and mouse expression data in refine.bio because we wanted to examine sex bias in data from both humans and a mammalian model organism. Mice have the largest amount of available expression data and are often used in drug development. We labeled a large fraction of these data; however, a small subset of samples could not be labeled due to platform challenges or poor quality expression data. Since this is a small fraction, and missing data is spread across many studies, we do not expect that labeling these data will change our conclusions; despite this, continued efforts will attempt to “rescue” these missing data. While we found only slight overall bias in publicly available mouse and human expression data, it is possible that public data for other organisms or proprietary data show different patterns of bias. In the future, we hope to extend our methods to sex label publicly available samples from other organisms with XY determination systems in order to further aid in the re-analysis and assessment of sex bias.

## Methods

The code used for this analysis is publicly available at https://github.com/erflynn/sl_label; all analyses were performed in R (3.6.1) or python (3.7.1);

### Dataset construction

#### Expression data pre-processing

We downloaded the normalized expression compendia and RNA-seq libraries for human (*Homo sapiens*) and mouse (*Mus musculus*) from refine.bio (3/15/2020). The compendia contain both microarray and RNA-seq data, quantile normalized and with missing values imputed using SVD-impute [50] (430,119 human, 228,708 mouse samples). We extracted the microarray data (330,508 human, 123,279 mouse samples) and converted the data to gctx format to aid analysis [51], no other transformations were applied to them. All RNA-seq libraries for human and mouse were downloaded from refine.bio (122,864 human, 125,652 mouse). RNA-seq samples with fewer than 100,000 counts were removed, resulting in 119,863 human and 121,828 mouse samples. Transcripts per million (TPM) counts were extracted from the salmon quant.sf files output [52]. To convert RNA-seq count data to a normal distribution for logistic regression, the data were transformed with the Box-Cox transformation using the R package BestNormalize [53].

#### Extraction of sample and study metadata labels

GEO microarray metadata were extracted from GEOMetadb [54], including study information (title, description, date), sample-study membership, and sample titles and attributes included in the *characteristics_ch1* field. Following a similar process to refine-bio metadata harmonization, RNA-seq metadata was extracted from European Nucleotide Archive (ENA) [55] XML files, including study information, study-run membership, run-to-sample mappings, and sample attributes. All runs were assigned the attributes of their corresponding samples (each sample can map to multiple runs but no run maps to multiple samples, for consistency with other data sources, we refer to SRA runs as “samples” from here forward. ArrayExpress microarray metadata was also extracted from ENA json files.

Sample metadata were present for 443,611 of 448,827 (98.8%) GEO samples, all 4960 ArrayExpress samples, and 237,267 of 241,691 RNA-seq runs (98.2%, corresponding to 196,852 unique samples).

### Sex labeling

#### Metadata sex label extraction

Sample-level sex labels were extracted from metadata sample attributes by filtering for keys that contained the words “sex” or “gender”. Additional attributes were also extracted if values contained exact matches to the words “male” or “female”. All unique values were then mapped to one of “male”, “female”, “mixed” sex (e.g. pooled sample from both males and females), or “unlabeled”. The human labels for the refine-bio RNA-seq samples almost exactly match those of MetaSRA [21] (30,063 of 30,073 samples).

We grouped studies into the following categories based on the provided sample sex labels:

1. *Unlabeled:* studies with either less than half of their samples labeled (for studies with up to sixty samples) or less than thirty samples labeled (for studies with more than sixty samples)
2. *Male-only:* all male labels
3. *Female-only:* all female labels
4. *Mostly-male:* > 80% of labeled samples are male
5. *Mostly-female:* > 80% of labeled samples are female
6. *Mixed sex:* ≤ 80% of labeled samples belong to either sex

To distinguish between studies with similar and highly imbalanced male/female proportions, we created separate categories for mixed sex (≤ 80%) versus mostly-male and mostly-female studies (> 80%). We selected a cutoff threshold of 80% because this roughly corresponds to dips in the distributions of study sex fraction (Additional file 1: Fig. S13). See Additional file 2: Tables S1A, B for the sample and study sex breakdowns respectively.

#### Inferring sample sex from expression data

##### Training and testing data

There is often substantial overlap of samples across studies, with groups of samples belonging to multiple studies. To reduce this overlap, we removed studies that share one or more samples with greater than five other studies. For the remainder of studies, we aggregated studies into study groups such that all samples that share any study are in the same study group.

For each organism and data type (human, mouse, and microarray, RNA-seq), we stratified by study group, sampled a maximum of five samples per study (to limit overfitting to a particular study), and then randomly selected training (n = 2,300–3,200 samples, 540–1,300 studies, ranges listed across four datasets) and testing data (n = 630–790 samples, 120–360 studies) such that the resulting datasets were approximately balanced between males and females (48.4–50.7%) (see Additional file 2: Table S3A for size of datasets by organism and data type). The goals in constructing these data sets were to ensure there was no leakage between training and test sets, limit overfitting, and retain sufficient samples and studies to perform stratified cross-validation.

##### Model features

We used X and Y chromosome genes as features for our sex labeling models. We extracted lists of human (GRCh38.p13) and mouse (GRCm38.p6) X and Y chromosome Ensembl transcripts and genes from biomaRt (version 2.40.5) [56] and X escape genes from the literature [29, 57, 58]. To examine the presence of potential technical artifacts in XY chromosome read mapping, we compared Y chromosome RNA-seq read counts in males and females, and found limited but present mapping of reads to the Y chromosome in samples labeled female (median 8.70, interquartile range (IQR) 3.62–21.0) versus much more prevalent mapping to the Y chromosome in males (median 268, IQR 86.1–505) (Additional file 1: Fig. S14).

##### Model selection and training

We compared the performance of a wide range of machine learning models, including penalized logistic regression methods (lasso, ridge, and elastic net) to K nearest neighbors (KNN), Support Vector Machines (SVM), Linear Discriminant Analysis (LDA), Naive Bayes, Random Forest, AdaBoost, Decision Trees, and a Multi-layer perceptron (MLP) [using scikit-learn version 0.24 [59]]. Model performance was assessed with six-fold cross-validation. Across the four datasets (mouse, human microarray and RNA-seq data), many methods have similar performance, specifically we see the highest performance from Random Forest followed by penalized logistic regression, MLP, and AdaBoost, which have similar performance (Additional file 1: Fig. S2 and Additional file 2: Table S2). While Random Forest shows the highest performance, given the similarly high performance and increased interpretability of penalized logistic regression, we use this method for the remainder of our analysis.

We trained logistic regression models with an elastic net penalty (using the R package glmnet version 4.0–2 [60]) using X and Y chromosome genes as input. We used nested cross-validation to select the hyperparameters alpha (elastic net penalty) and lambda (shrinkage parameter). Briefly, for each of the six cross-validation folds (study-stratified), each value of alpha (0.1 to 1 in increments of 0.1), lambda was selected from a grid of values by performing cross-validation on five of the six folds and selecting the lambda within one standard error of the minimum mean cross-validated error (“lambda-1se”). This model was assessed on the sixth “validation” fold. We then computed the median classification error and median lambda for each alpha across all six validation folds, and selected the value of alpha (and its corresponding lambda) with the lowest median error.

We performed nested cross-validation in this way because of the substantial between-study heterogeneity and within-study correlations in expression data. Selection of hyperparameters without the additional cross-validation loop led to increased classification error. The procedure described is equivalent to the percentile lasso [61] using the 50th percentile, and extended to select both alpha and lambda.

##### Accuracy assessment and cutoff selection

We assessed the accuracy of our model using both the held-out test set (described above) and an extended test set consisting of all samples with metadata sex labels from studies not in either the training set. We consider a sample to be correctly labeled when metadata sex matches the expression-based sex; however, it is important to note that we are actually measuring concordance, as metadata sex labels may contain errors. We also examined two subsets of the extended test set: samples from large (1) *mixed sex* studies (at least 10 samples per study) or (2) *exclusively single sex* studies (at least 8 samples per study, with all samples present and annotated as from one sex) and assessed the accuracy on these subsets of the data (see Additional file 2: Table S3A for a full list of assessment datasets and their sizes, and Additional file 2: Table S3B for the corresponding accuracies).

We created the exclusively single sex datasets for two reasons: (1) we expect that if metadata indicates all samples in a study are of one sex it is less likely there are misannotation errors, and (2) to make sure that within study variability did not skew or ability to label these data. In microarray data, in particular, because these data are signal intensities rather than counts, improper normalization of single sex studies can lead to a wider distribution of sex relevant gene expression values.

For assigning sex labels to samples, we used a cutoff threshold of 0.7 on the model predicted probabilities in order to approximate 95% accuracy (see Additional file 1: Fig. S3). This leads to labeling of 92.8% of the human microarray and 72.3% of the human RNA-seq extended test sets (see Additional file 2: Table S3B for mouse statistics).

##### Assessment of performance across platforms

Platform heterogeneity presents a huge challenge for examining microarray data, and as a result, previous sex labeling methods have been limited to specific platforms. With our method, we aimed to have high performance across a range of platforms. Our models show high performance in the majority of platforms; however, they perform particularly poorly in a small number of platforms (less than 70% accuracy in either mouse or human) but these cover < 3% of samples (Additional file 1: Fig. S4 and Additional file 2: Table S4A, B). As a result, these seven platforms were excluded from subsequent analysis.

### Cell line sex label assessment

#### Cell line labeling.

##### Cell line normalization

Cell line names, synonyms, sex labels, and amelogenin Short Term Repeat (STR) marker results were extracted from Cellosaurus [33] (3/27/2020). We filtered for human and mouse cell lines (n = 109,426 unique accessions). Cell line names and synonyms were converted to lowercase. Where multiple different accessions have the same name (n = 31 names, 760 synonyms) or the same names with different punctuation (n = 294 names, 579 synonyms), we map every instance to all accessions. If a synonym matches a cell line name it does not share an accession with (n = 412 exactly, 91 with different punctuation), we map that name only to the cell line accession where the name belongs, but not to both. A subset of the identically named cell lines have the same parent cell line (n = 77 names, 739 synonyms), in this case, the parent cell line is used for the subsequent analysis steps.

We mapped samples to cell lines by matching values to Cellosaurus names and synonyms. We performed mapping using three sets of attributes, of decreasing specificity: (1) attribute pairs with keys containing “cell” and “line”, (2) attribute pairs with values containing “cell” and “line”, and (3) attribute pairs that mention the word “cell”. Human and mouse data were mapped separately, using the appropriate Cellosaurus subset. Prior to analysis, mouse strain names, common stop words, and cell line names/synonyms that consisted of all numeric characters were removed. We first used exact matches between the value and cell line names (≥ 3 characters) for attribute pairs with a cell line key. Then, cell line mentions were detected using n-gram matching (n = 1, 2, 3) between the attribute value (> 3 characters) to a cell line or synonym.

##### Labeling sample source type

Based on the presence of exact lexical matches to key terms in the sample metadata, we automatically assigned samples to one of: tissue, stem cell, xenograft, cancer cell, cell line, primary cell, or other. Cell line data was divided into “named” and “unnamed” cell lines, where named cell lines map to a Cellosaurus identifier (see Additional file 1: Fig. S15 for logic and Additional file 2: Table S10 for counts by sample type).

#### Examining cell line sex

Using our normalized cell line labels, we can compare the reference sex of a cell line from Cellosaurus to the inferred sex from our expression data. For this analysis, we examined cell line samples that were both labeled as a cell line using our expression model and mapped to a Cellosaurus ontology label based on its metadata.

Two types of reference sex labels from Cellosaurus were compared to imputed sex labels from our model; these include *donor* sex, the sex of the donor the cell line was derived from, and *recorded* sex, which is the sex of cell line samples derived from Short Tandem Repeat (STR) profiling of the amelogenin genes [39]. Samples are labeled as “both” if multiple STR analyses have obtained different sex labels.

For cell lines with corresponding data in the Cancer Cell Line Encyclopedia (CCLE) [31], we compared our sample sex scores to CCLE X and Y chromosome copy number (CNV) data (downloaded 9/5/2020).

### Estimation of metadata misannotation

We examined metadata sex label misannotation rates in three ways, described below.

#### Comparing mismatch rates in single sex versus mixed sex studies

We examined the rates of sample and study sex label mismatches in large single and mixed sex studies (≥10 samples). A sample mismatch is a sample with a metadata sex label of male or female and an expression-based sex label (with score above 0.7) indicating the opposite sex. A mismatched study means that the study contains at least one mismatched sample. We used a chi-squared test to examine whether the fraction of mismatched samples and studies was significantly different across mixed versus single sex studies.

#### Comparing expression-based methods in mixed sex studies

For large mixed sex studies (at least 5 male and 5 female samples), we compared metadata labels to expression labels predicted from the clustering-based methods in Toker et al. [20] and massiR [24] and those from our own classification method. We conservatively labeled a sample as a mismatch if all of the expression labels disagreed with the metadata label.

#### Clustering to identify high confidence swaps in mixed sex studies

**Fig. 5:**
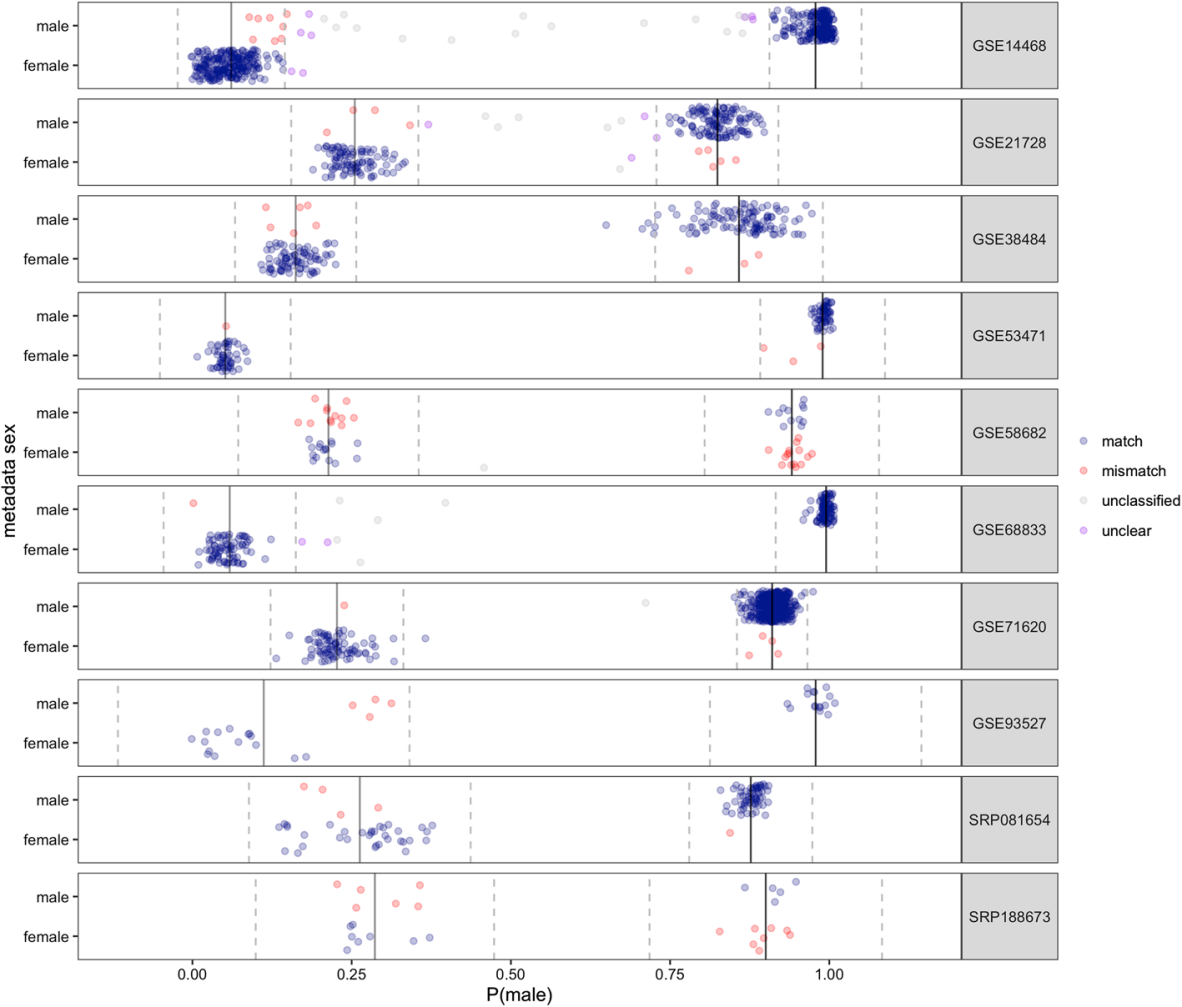
Leveraging within-study distributions of sample sex scores to identify high-confidence mislabeled samples. Each row is a study (randomly sampled from the list of mixed sex tissue studies with multiple clusters). Samples are separated by metadata sex (on the y axis) and our model sample sex score (P(male)) (on the x axis). Samples are colored by whether they show a high confidence (as indicated by a P(sample belongs to cluster) > 0.95) “match” (blue) or “mismatch” (red) between the metadata and expression-based sex; samples that were not classified by the model are labeled “unclassified” (gray), classified samples that do not pass the 0.95 threshold for their cluster are labeled “unclear” (purple). Clustering was obtained by fitting a mixture of Gaussians; and the estimated mean (solid line) and 95% confidence interval (dashed line) for each cluster is shown

While the model predicted probability (*“sample sex score”*) provides an estimate that a sample is a particular sex, we find there is a lot of study-to-study heterogeneity in the distribution of these sample sex scores. By clustering the sample sex scores, we can leverage information about their distribution within a mixed sex study to obtain a probability estimate that a sample belongs to either the male or female cluster (Fig. 5). This provides a more local estimate of mis-annotated samples at a study level and allows us to identify high confidence swaps. To obtain a probability estimate that a sample belongs to a cluster, we fit a mixture of one-dimensional Gaussians.

To identify high confidence misannotated samples in mixed sex studies, we used Gaussian model based clustering (R package Mclust [62]) to cluster the sample sex scores within a study. We performed clustering by fitting a mixture of Gaussians using the unequal variances model. We used the default prior with a larger scale parameter (scale = 0.15) to account for the spread of samples. Noise was added in the case that sample sex scores fell in the unclassified category (0.3 < *p*(male) < 0.7) and was initialized to the set of these samples; however, for studies with $$> \,{\raise0.7ex\hbox{$1$} \!\mathord{\left/ {\vphantom {1 3}}\right.\kern-\nulldelimiterspace} \!\lower0.7ex\hbox{$3$}}$$ samples unclassified, we did not include noise terms to help with convergence. The number of Gaussians (n = 1 or 2) and the best model for each study was selected using Bayesian Information Criterion (BIC). We additionally filtered studies with little separation between clusters, removing studies where the difference in means between the two clusters’ sample sex scores was less than 0.3 (n = 2 studies). For the remaining studies, we set a cutoff posterior probability of 0.95 for assignment of a sample to a cluster, in cases where the metadata sex does not match that cluster, we have a “mismatched” sample. The remaining samples are labeled “unclassified” (if the model estimated that they were noise) or “unclear” (assigned a cluster by the model but with probability < 0.95).

### Sex bias in drug data

#### Drug labeling

##### Study drug mention labeling

Studies were labeled as having a drug mention if the metadata contained a drug name. DrugBank [63] (date accessed: 4/14/2019) XML data was downloaded and synonyms and drug names parsed. Names or synonyms 3 or fewer characters long were discarded, as well as common stop words. Then, we used n-gram matching (n = 1, 2, 3) to map between the metadata text (either study or sample) and a drug or synonym.

To find drug-containing studies with high sensitivity, we labeled studies based on *drug mentions* in their metadata. Study mapping used the text from study title and description fields. Out of 44,184 total studies, 7665 (17.3%) contained a drug mention (1104 drugs).

##### Sample-level annotation of drug labeling

To build a high specificity dataset, we also performed sample level labeling using the refine.bio harmonized “treatment” and “compound” fields, as well as the sample title.

We created a library of common control terms for sample mapping. This vocabulary consists of the following words:

"none", "control", "untreated", "dmso", "na", "placebo", "saline", "pbs", "mock", "baseline", "unstimulated", "etoh", "ethanol", "ctrl", "non-treated", "vehicle", "ctl", “no treatment”

We then identified the subset of studies where the drug mentioned in a treatment field is the drug mentioned in the study; we call these *drug exposure studies*.

#### Assessment of sex bias in drug data

Anatomic Therapeutic Class (ATC) drug mappings were extracted from DrugBank. The enrichment of male only versus female only, and single versus mixed sex studies in each class was assessed separately using chi-squared tests. Prior to running tests, we removed classes with less than 5 samples in a category. We filtered for a chi-squared *p *value < 0.05/48 ≅ 0.001 using a 557 correction on the number of tests (n = 48). We also grouped by drug and calculated the fraction of male-only and female-only studies for each drug.

## Supplementary Information

**Additional file 1.** Supplementary Material and Figures.

**Additional file 2.** Supplementary Tables.

## Data Availability

All data analysed during this study was previously publicly available through GEO, SRA, and ArrayExpress. Generated cell line annotations of potential Y chromosome loss and drug-study mappings are included in the supplement. In addition, the sample sex, source, drug, and cell line labels will be made publicly available through refine.bio (https://www.refine.bio/) to allow researchers to examine these labels and use them to better search for and re-analyze existing studies. All generated data and labels are also available through the figshare repository, https://figshare.com/s/985621c1705043421962. The code used for labeling and analysis is publicly available at https://github.com/erflynn/sl_label.
