# Supplementary material for: Large-scale labeling and assessment of sex bias in publicly available expression data
Source: BMC Bioinformatics. 2021 Mar 30;22:168. doi: 10.1186/s12859-021-04070-2 (PMC8011224; doi:10.1186/s12859-021-04070-2)
Supplement: Supplementary file 1 — Additional file 1. Supplementary Material and Figures. [file 12859_2021_4070_MOESM1_ESM.docx]

**Supplementary Material** for *Large-Scale Labeling and Assessment of Sex Bias in Publicly Available Expression Data*.

Authors: Emily Flynn, Annie Chang, and Russ B. Altman

**SUPPLEMENTAL TABLES**

**Supplementary Table 1.** Sex breakdown by sample (A) and study (B). These data are divided by organism, data type (microarray or RNA-seq), and label type (“metadata” or “expression”), where expression refers to the labels from our model. The sample-level table includes counts of male, female, and unlabeled samples, while the study-level table includes the counts of studies in each of the study types (see Methods 2-1). Fractions of samples and studies are also included.

Supplementary Table 2. Performance of across machine learning methods. Each column is a dataset (human or mouse, microarray or RNA-seq), and each row is the result from training a different model. The values shown are the median agreement across training data folds.

**Supplementary Table 3.** Accuracy assessment. A) List of assessment datasets for each organism, data_type, and the number of studies and samples and sex breakdown of each dataset. B) Accuracy of sex labeling in each assessment dataset at varying “cutoff” probability scores.

**Supplementary Table 4.** Platform accuracy. A) List of all platforms, the number of samples of that platform, and accuracy and fraction of samples from that platform labeled by our model (at cutoff threshold 0.7). B) Fractions of all samples belonging to platforms with poor accuracy (“poor_accuracy”, defined as <70% concordance), not included in the extended test set (“not_in_test”), and included samples after removing poor accuracy or untested platforms (“included”). The approximate accuracy (“approx_accuracy”) is calculated by multiplying the accuracy of that platform by the prevalence of the platform overall, and the approximate fraction of samples labeled (“approx_frac_labeled”) is calculated by multiplying the fraction labeled in platform a platform by the prevalence of the platform. These refer to the accuracy and fraction labeled in the included set.

**Supplementary Table 5.** Model coefficients and X inactivation status in human microarray (A), human RNA-seq (B), mouse microarray (C), and mouse RNA-seq (D) models. The gene name, ensembl gene (and transcript for RNA-seq) identifiers, chromosome name, region (PAR or non-PAR), and literature X chromosome inactivation status (with associated references) are listed for each coefficient. Human homologs are given for mouse genes.

**Supplementary Table 6.** Y chromosome loss across 87 male cell lines. Includes the cell line name (“cl_name”) and accession, number of samples and studies, number of samples labeled male and female, and the median P(male) across all samples (“median_p_male”)

**Supplementary Table 7.** Misannotation estimates in (A) single versus mixed sex studies, (B) mixed sex studies by comparison to other expression-based methods, and (C) mixed sex studies based on the distribution of sample sex scores. Estimates are given at varying thresholds of our model (A + B) or for clustering cutoffs (C).

**Supplementary Table 8.** (A) List of drug exposure studies. Each row is a study/drug pair, and includes the DrugBank ID (“dbID”), “drug_name”, and ATC code. The “sample_terms” column refers to whether the study contains samples with both control and drug terms (“both”) or only drug terms (“drug-only”). (B) Sex bias by drug. Each row contains the organism, drug (with DrugBank ID, name, and ATC code), number of studies, and fractions male, female, and mixed sex studies.

**Supplementary Table 9.** Studies with cancer (A) (ATC class L) and nervous system drugs (B) (ATC class N), organized by drug and study sex breakdown.

Supplementary Table 10. Counts by sample source type, organized by the number of samples assigned to each source type.

**SUPPLEMENTAL FIGURES**


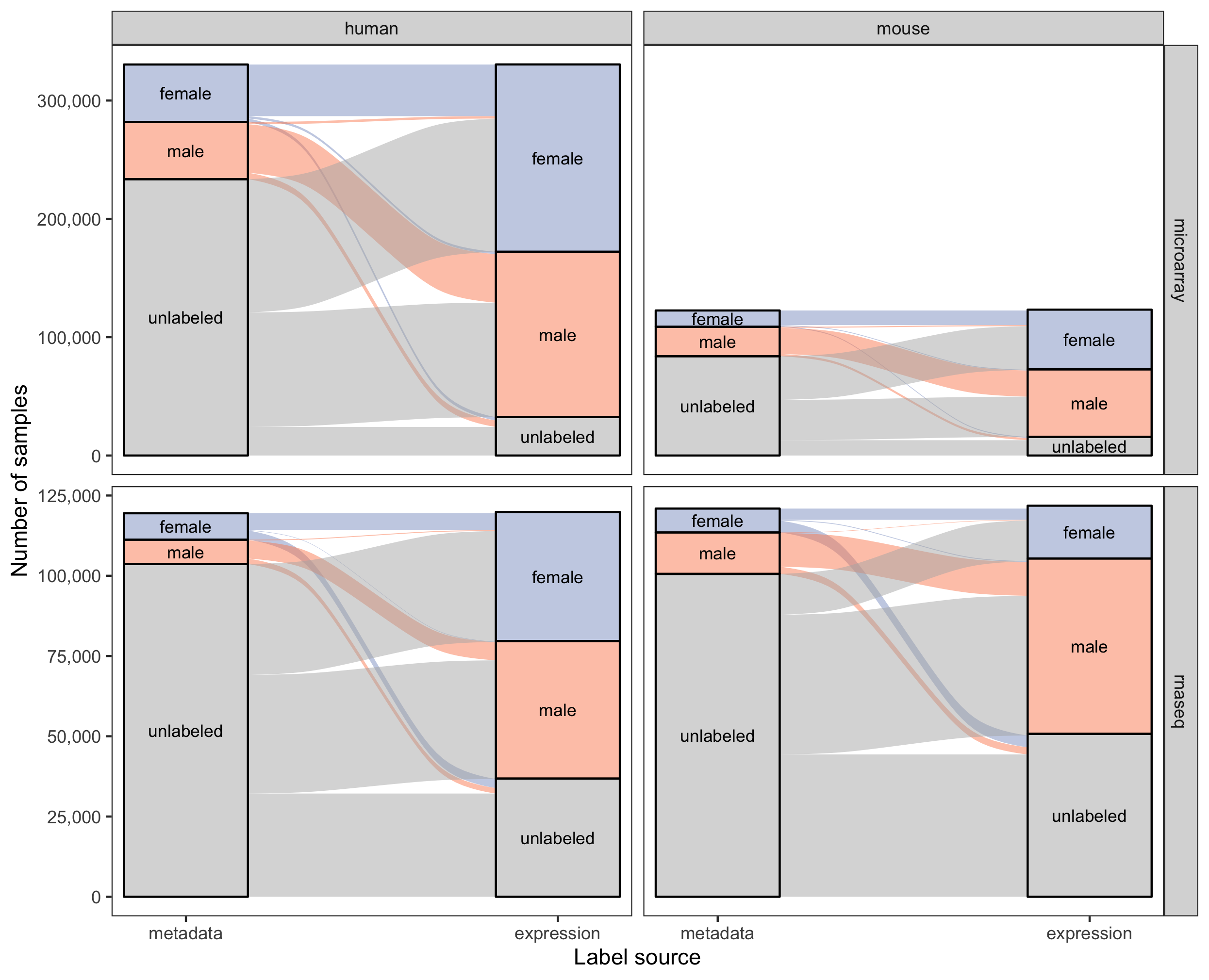


**Supplementary Figure 1.** Breakdown of sample sex labels. Same as Figure 2 but at a sample rather than study level.


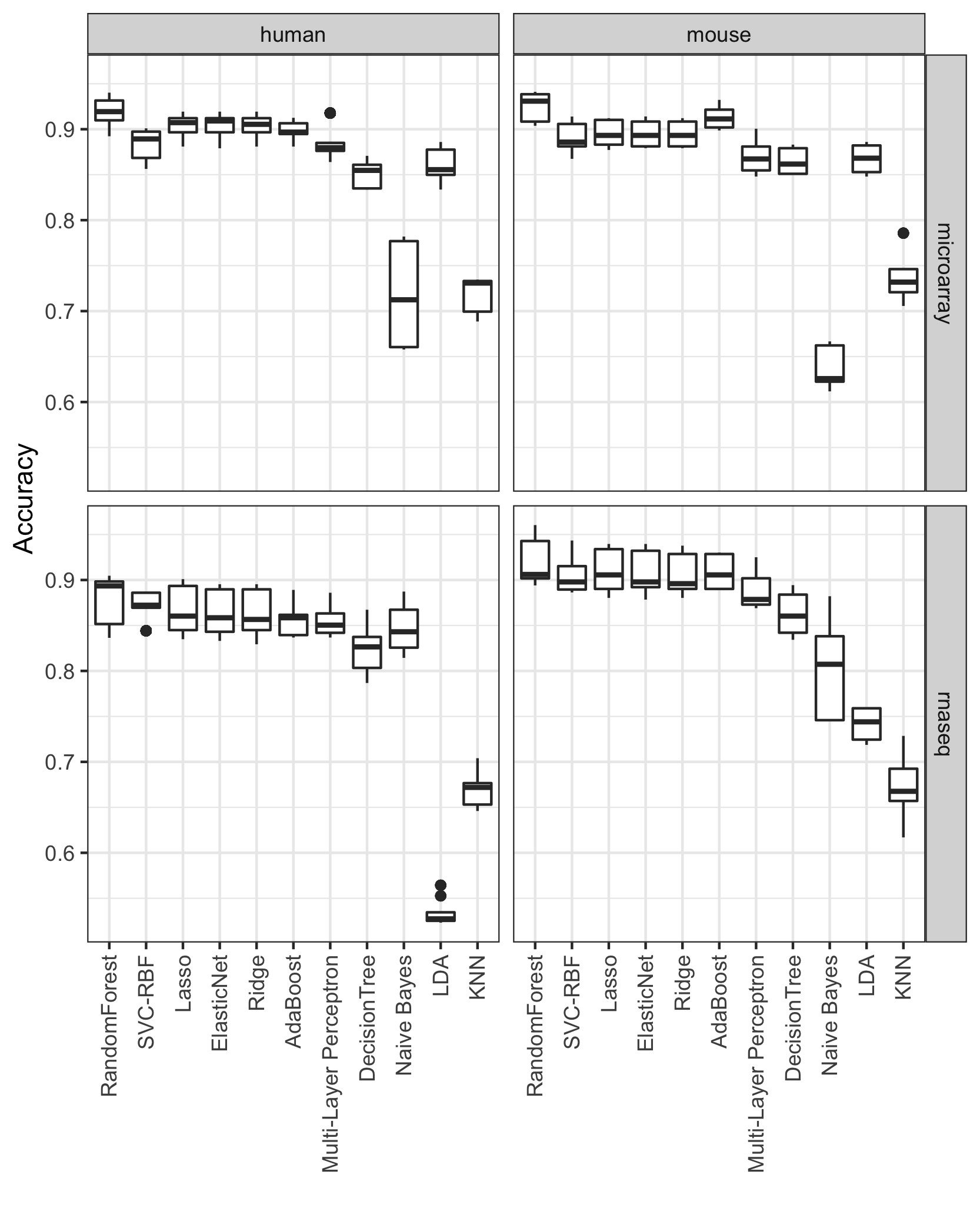


**Supplementary Figure 2.** Comparison across machine learning methods. Accuracy across six-fold cross-validation using the training set.


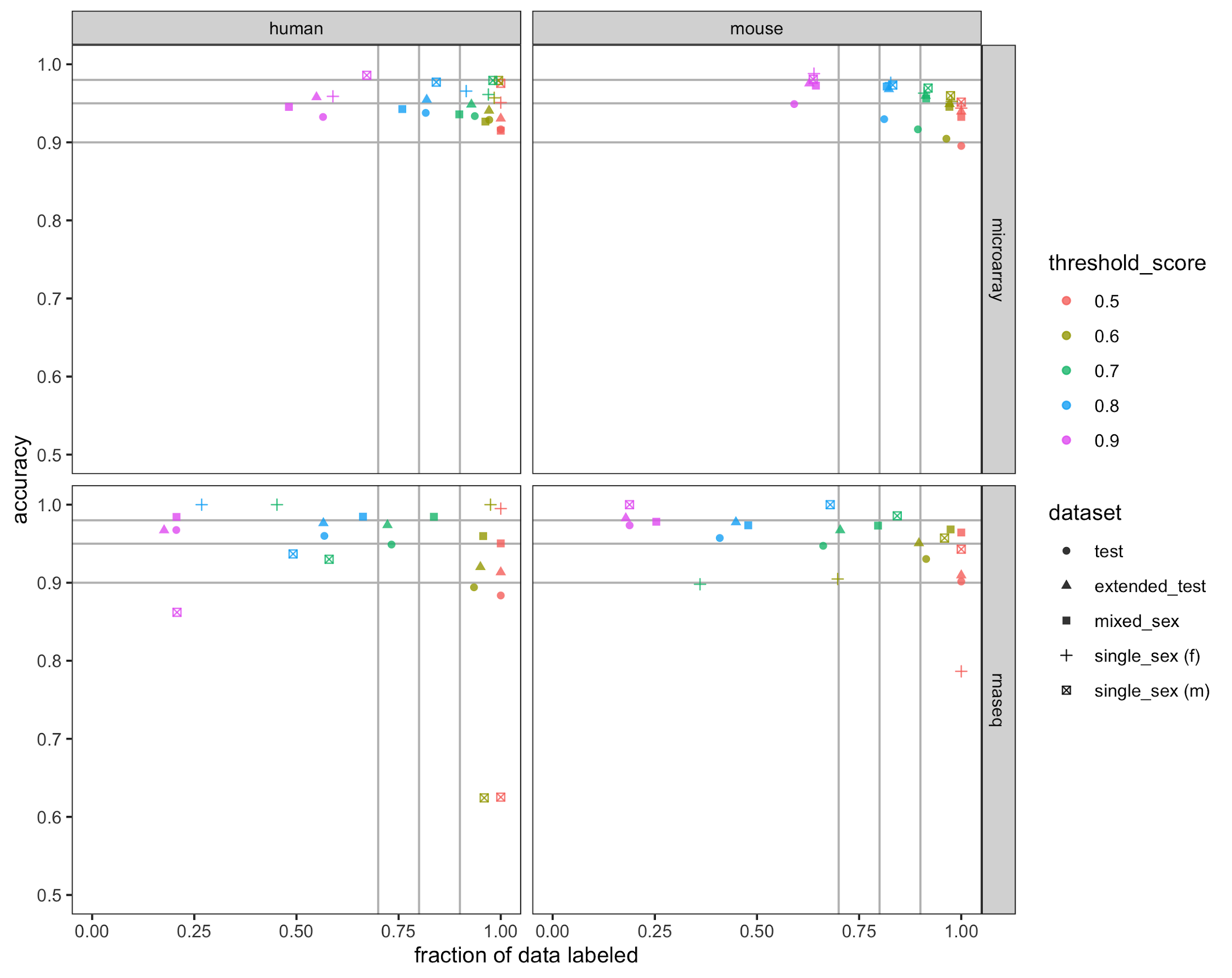


**Supplementary Figure 3.**  Accuracy and fraction of data labeled at certain sample sex score cutoffs. Each color is a different sample sex score cutoff, the shape of the point indicates the accuracy and fraction labeled on the dataset it is being evaluated on. Gray lines highlight the 0.9, 0.95, 0.98 accuracy cutoffs and 0.7, 0.8, and 0.9 data fractions.

A)
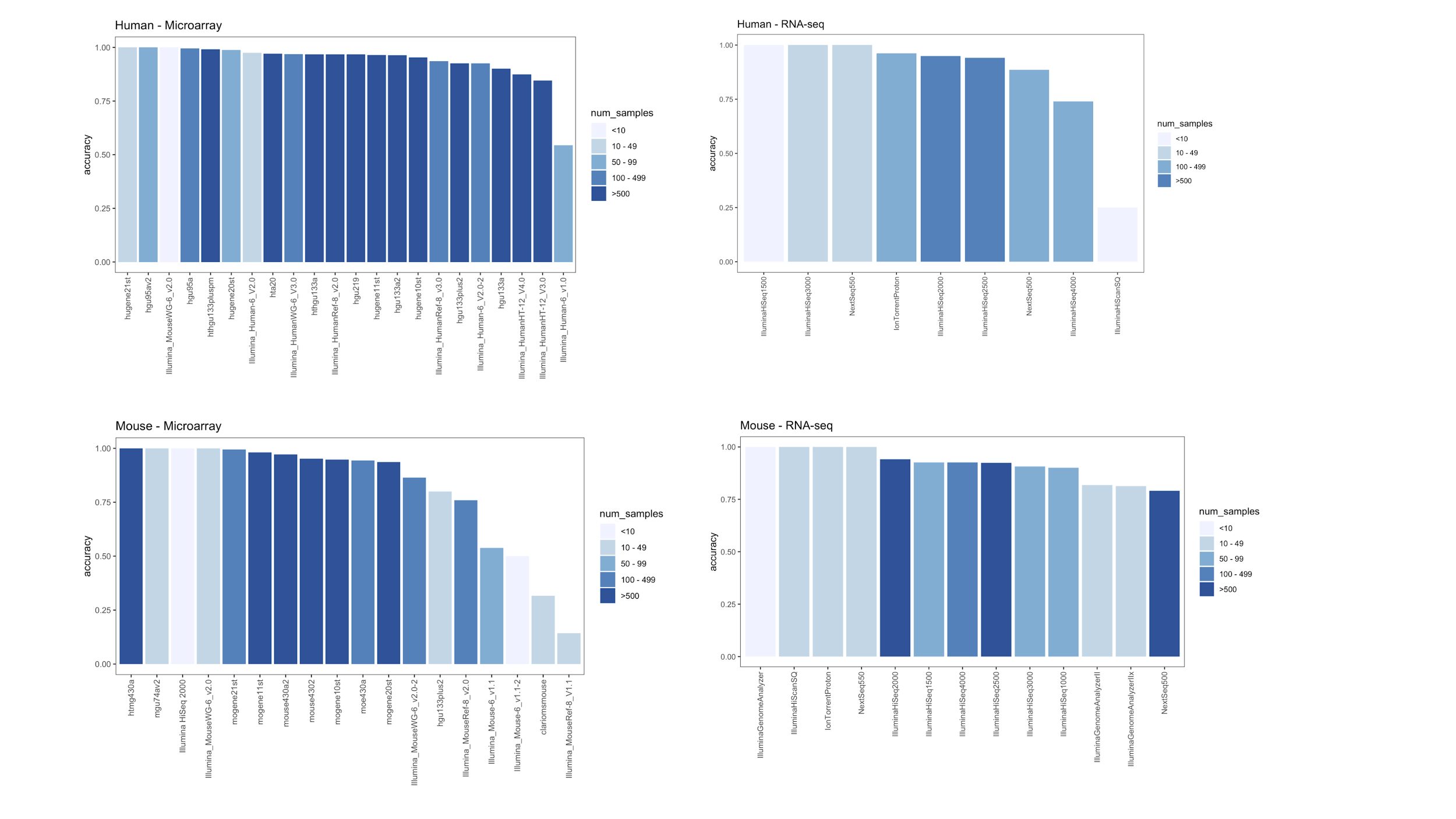
B)
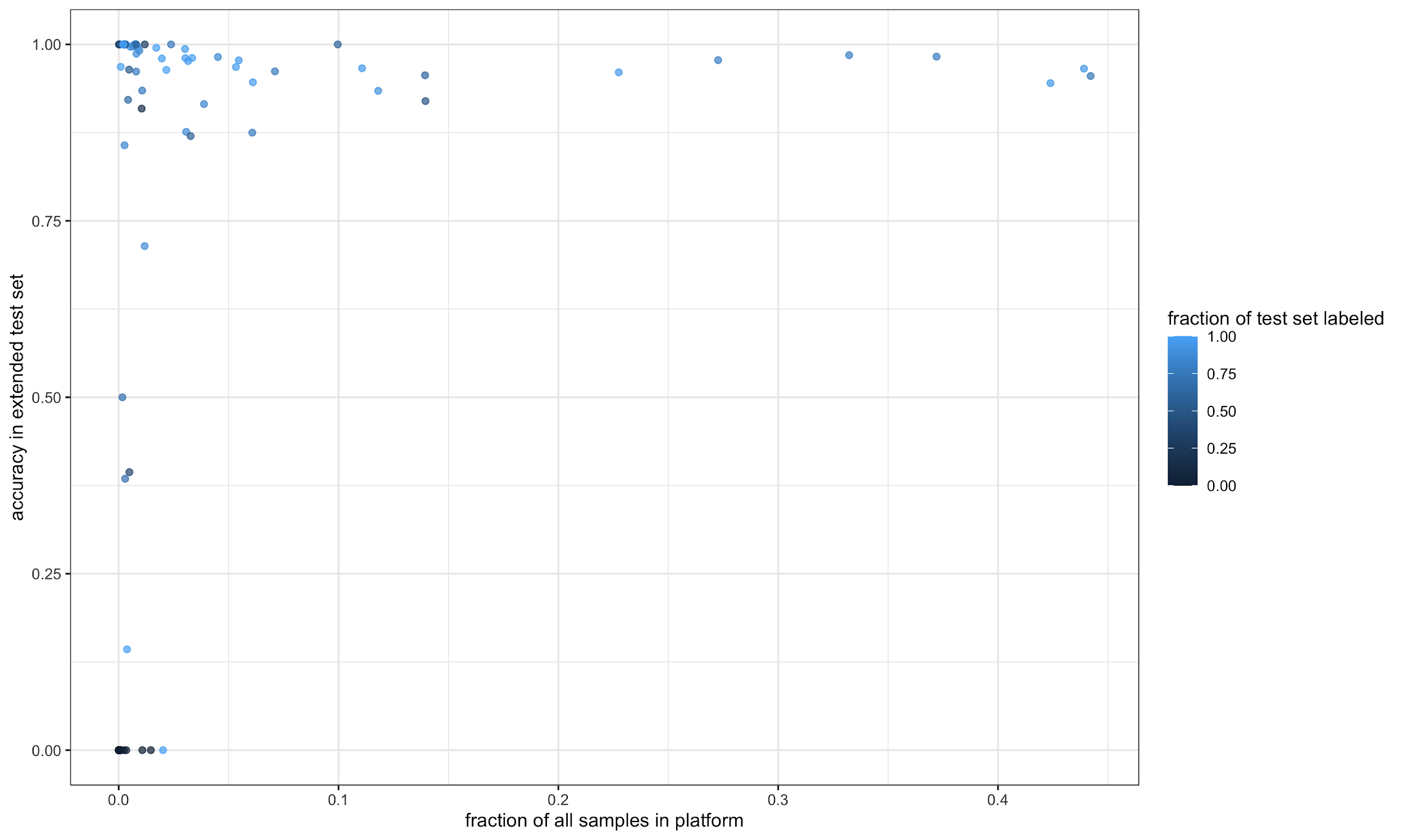


**Supplementary Figure 4.**  A) Accuracy across platforms. Platform (Illumina MouseRef8 Ver1.1) which has 0% accuracy in the extended test set is not shown in these plots. B) Platform accuracy in the extended test set versus the fraction of all samples in that platform, where each point is a platform. Bars and points are colored by the number of samples in that platform.

A)
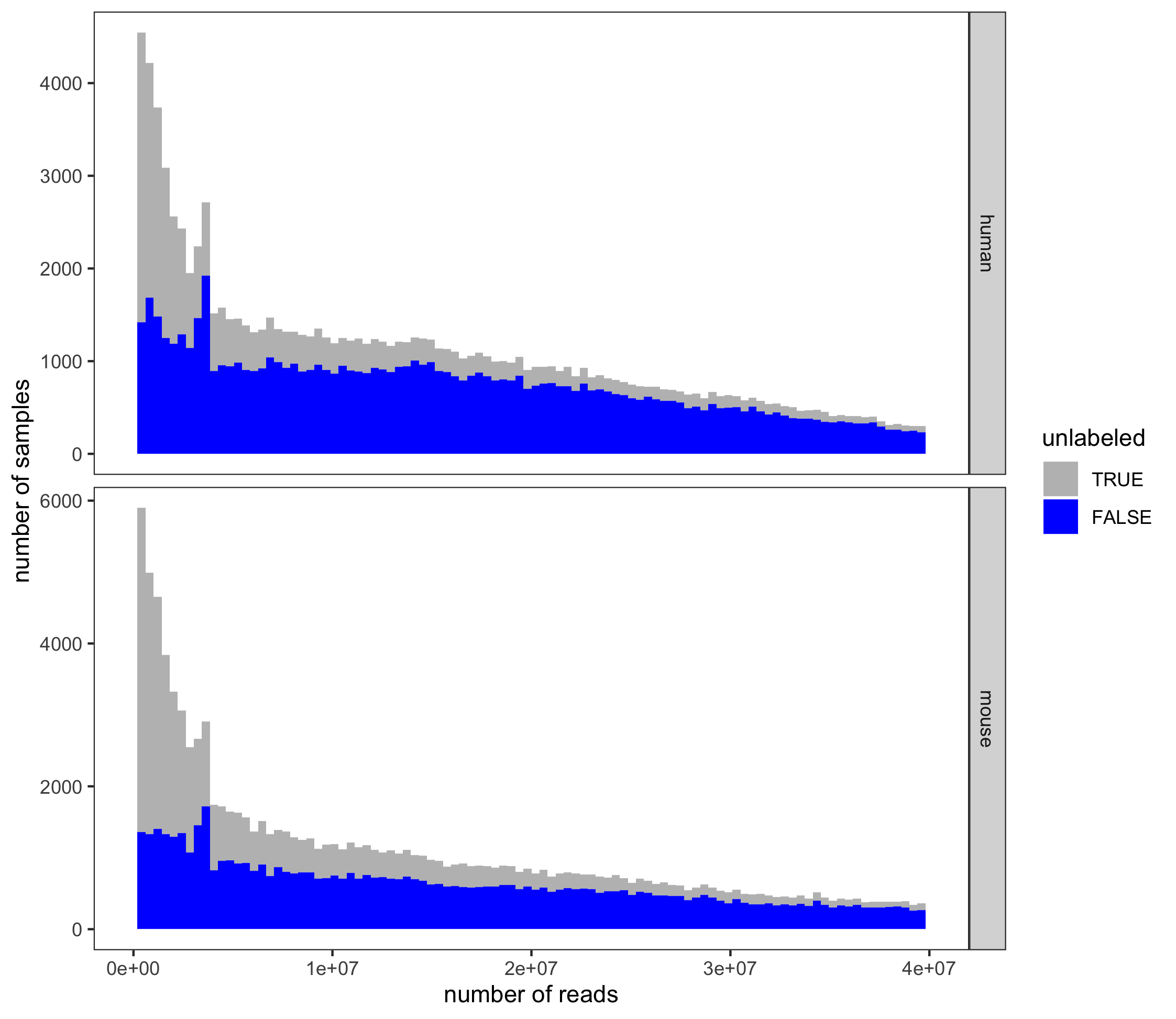

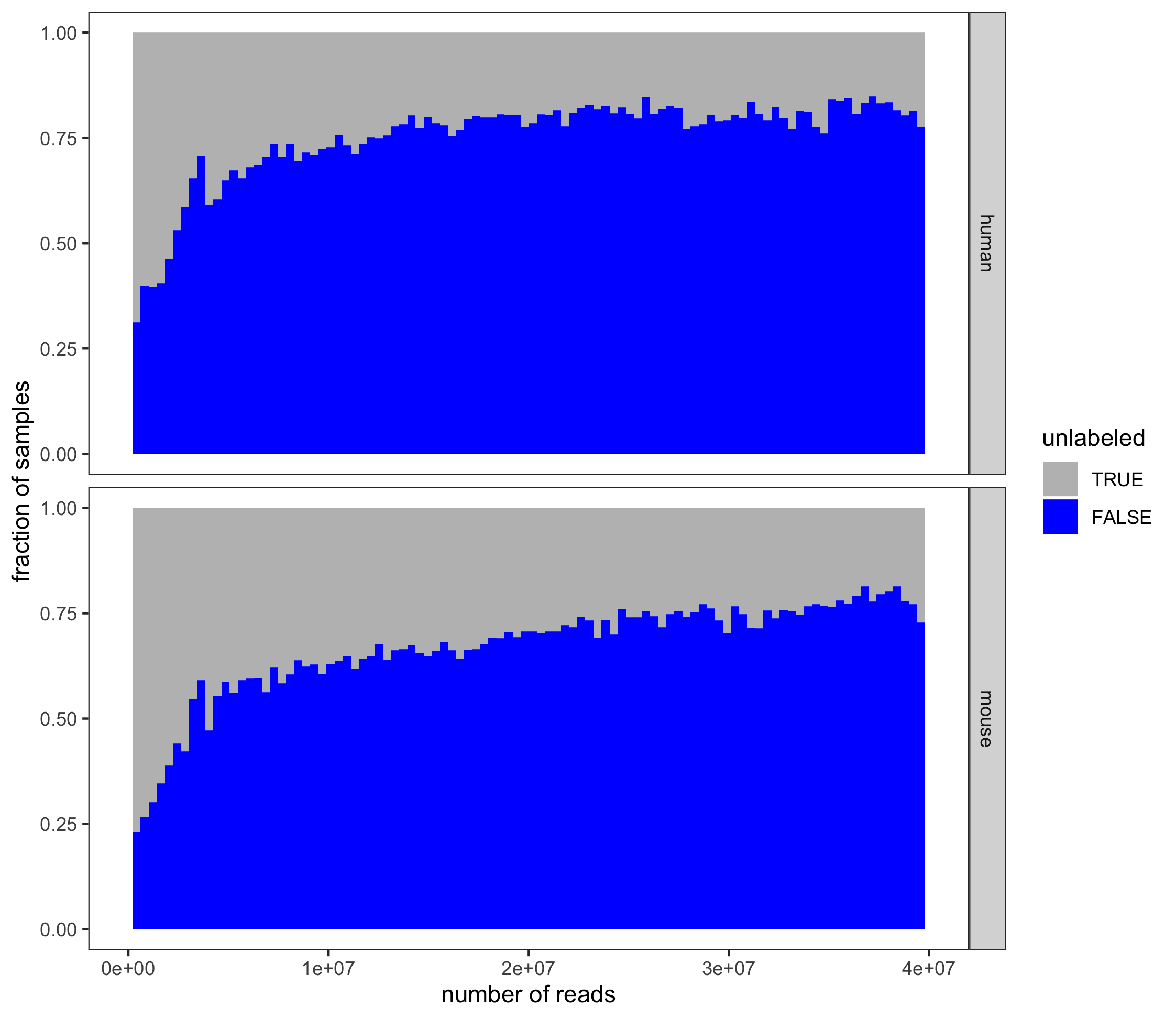


B)
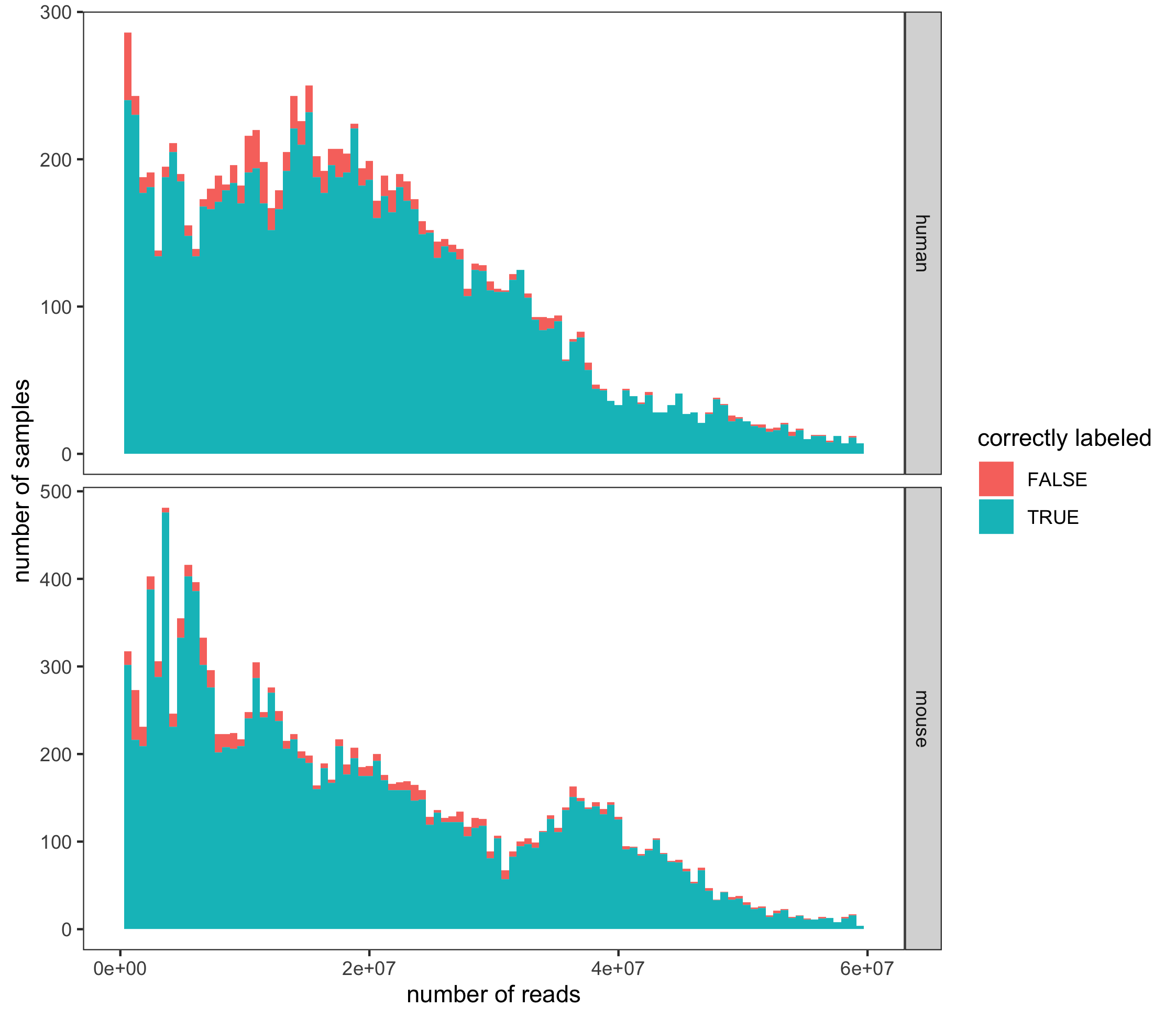

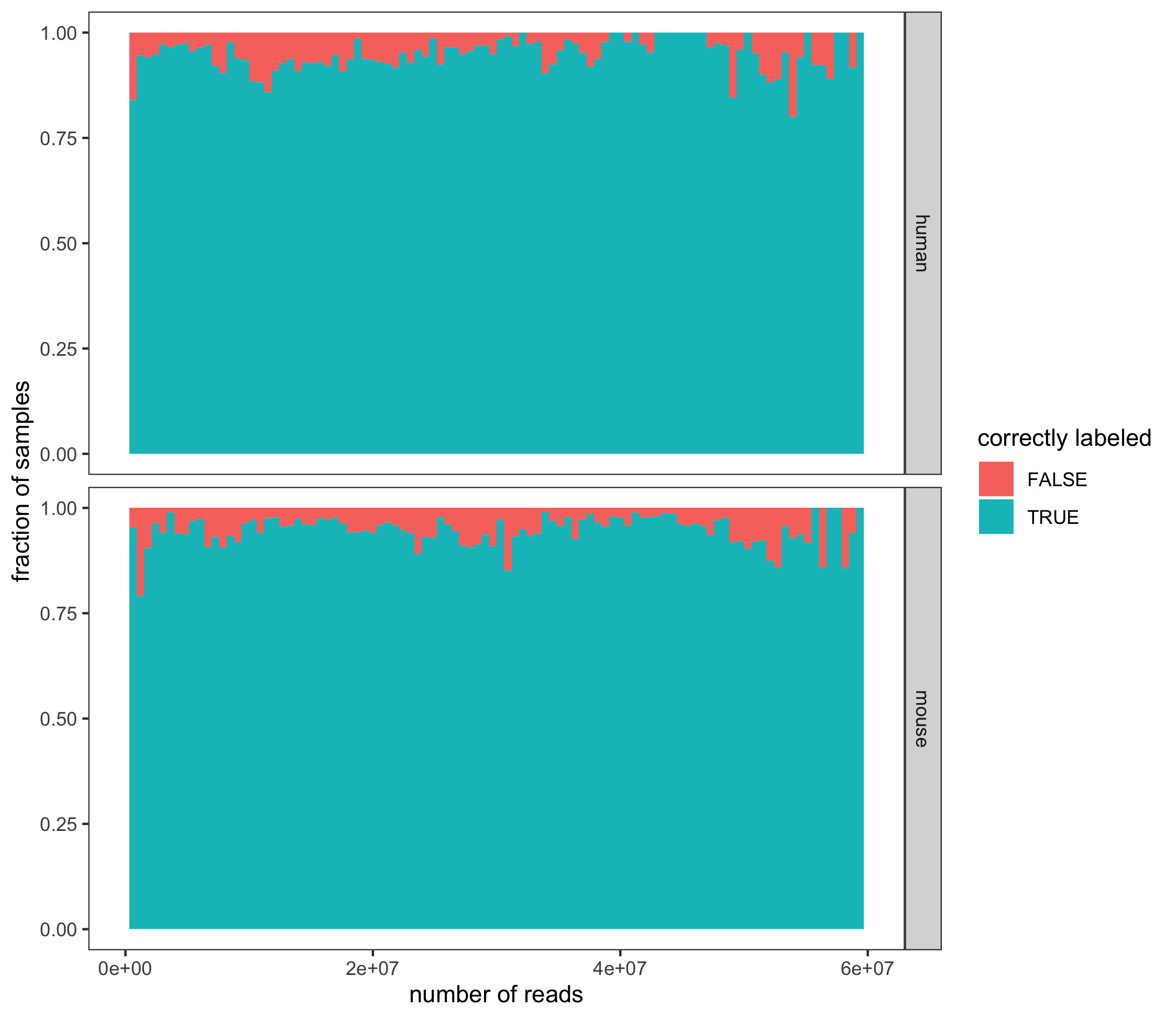


**Supplementary Figure 5.** RNA-seq counts and labeling. A) Our RNA-seq models label a larger fraction of RNA-seq samples with more reads (blue indicates labeled samples, gray indicates unlabeled samples). B) For samples with metadata labels, we see a limited relationship between number of reads and agreement of expression and metadata labels (correct labeling turquoise, incorrect labeling in red). Both the numbers of samples (left) and fraction of samples (right) in each category are shown.

A)
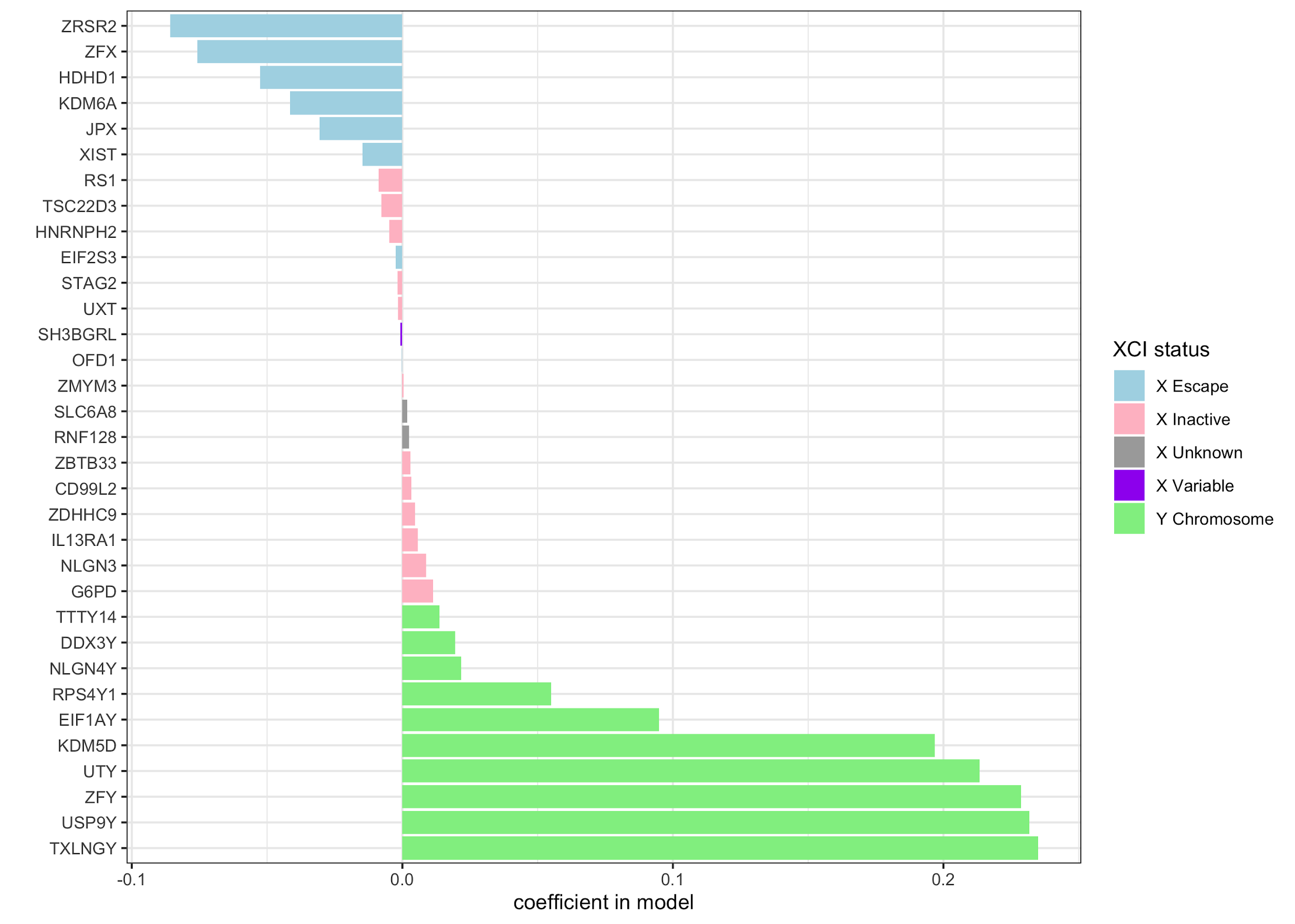
B)
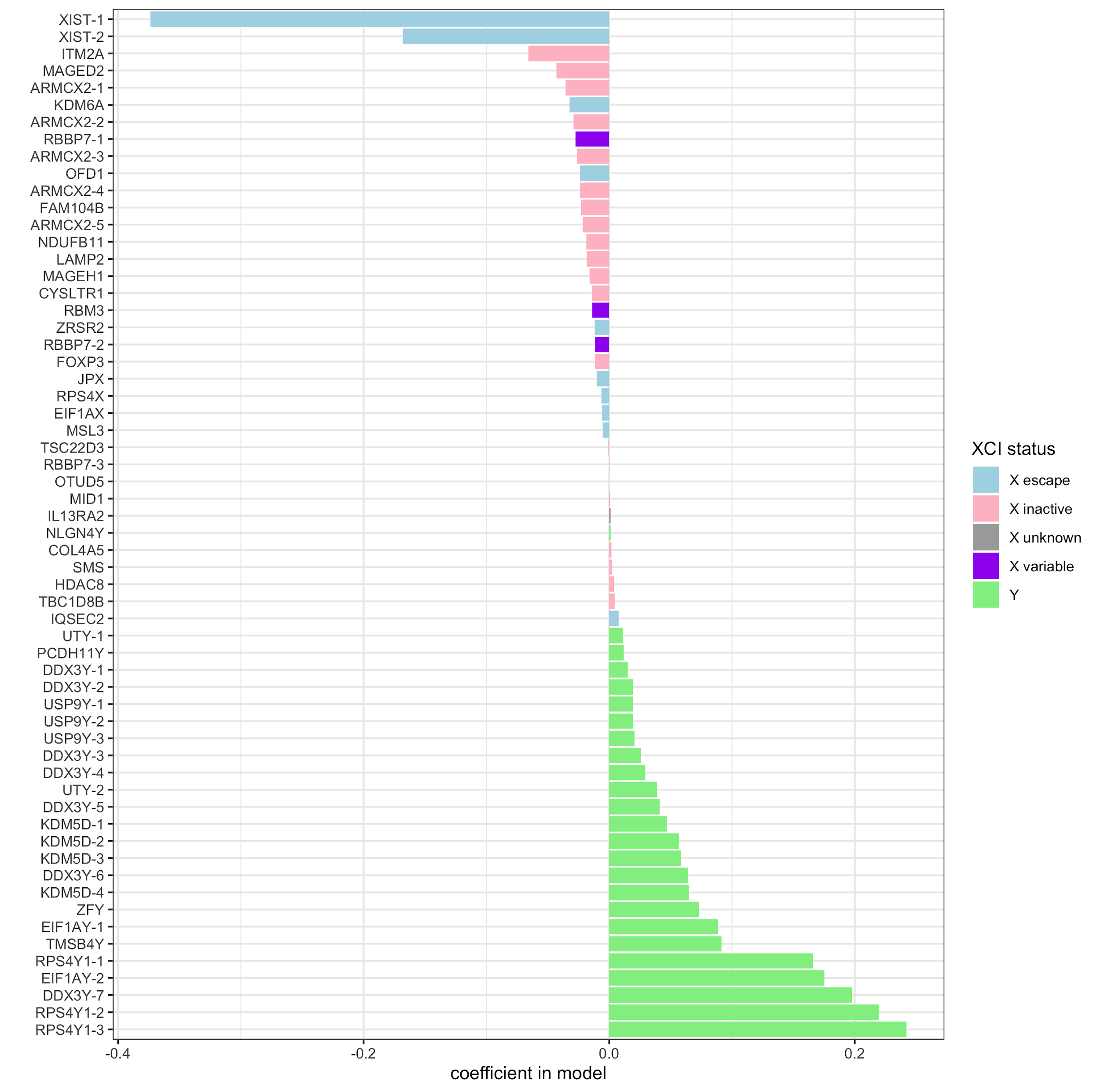

C)
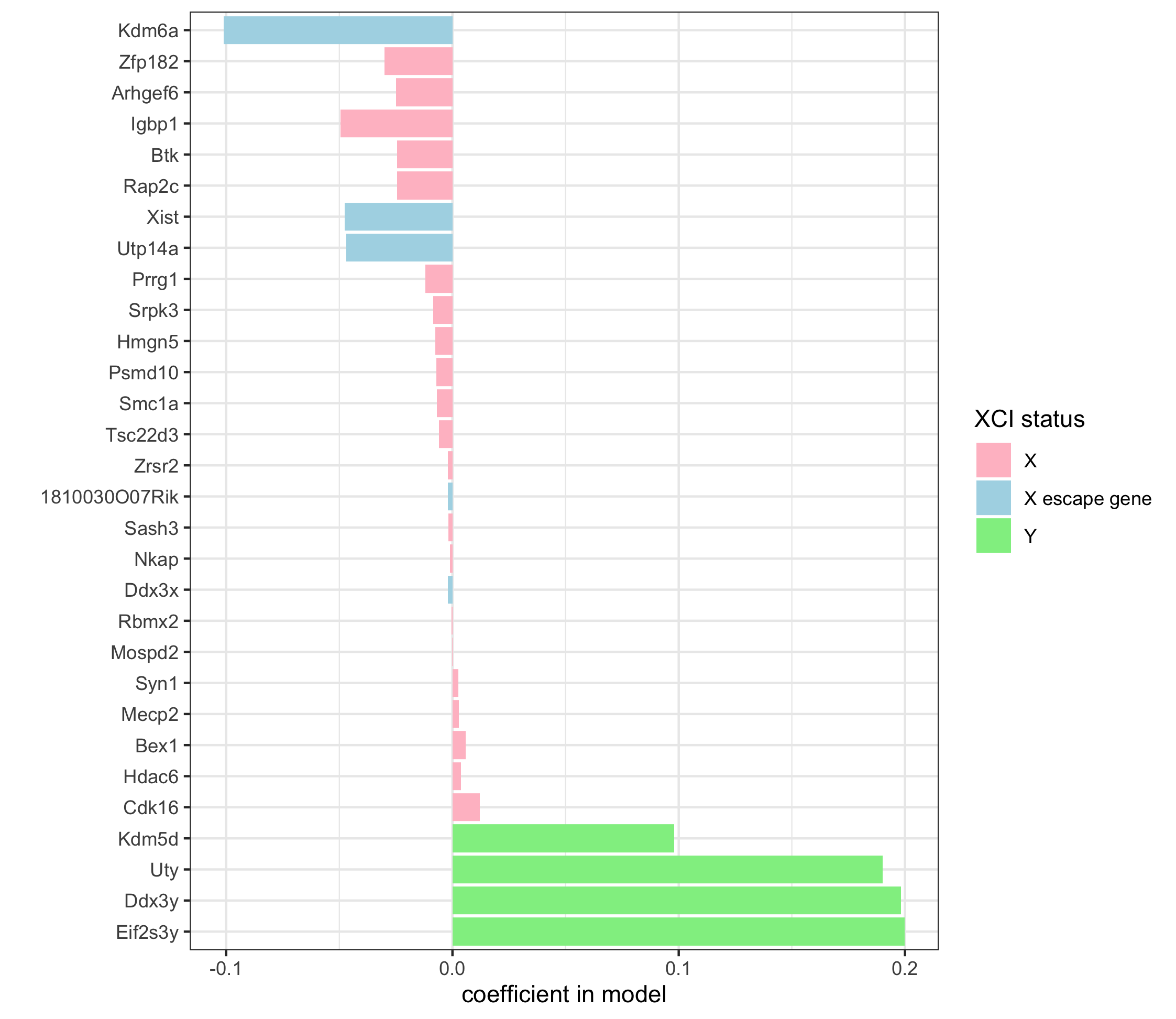
D)
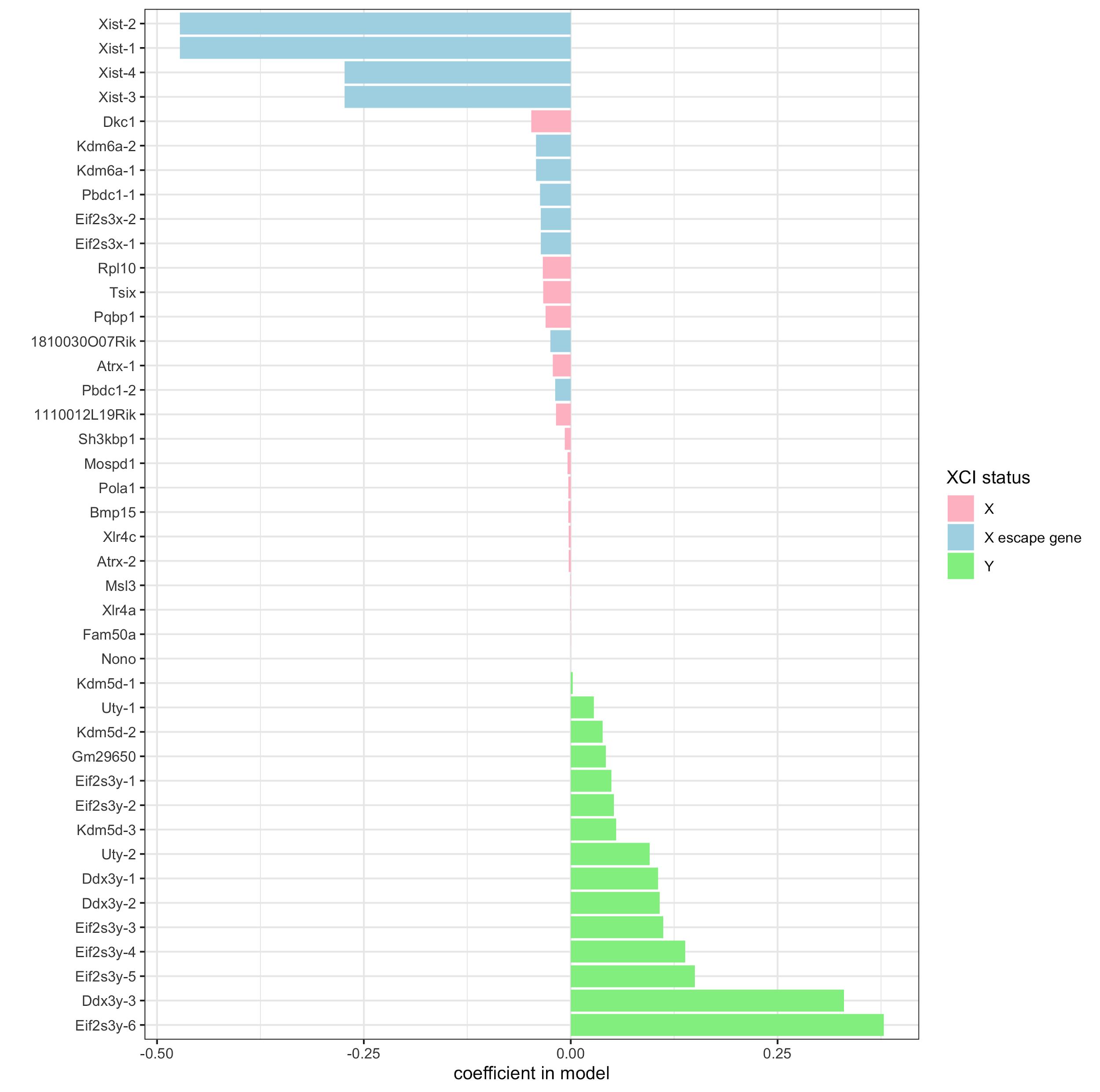


**Supplementary Figure 6.** Model coefficients and X inactivation status in human microarray (A), human RNA-seq (B), mouse microarray (C), and mouse RNA-seq (D) models. The values of the coefficients are plotted, colored by the literature X inactivation status [(from Yang et al. 2010 and Berletch et al. 2015 for mouse;](https://paperpile.com/c/g7uvfG/Acqb+QfEn) [Tukiainen et al. 2017 for human)](https://paperpile.com/c/g7uvfG/OKY1), where blue is escape from inactivation, pink is inactivation, purple is variable inactivation, and gray is unknown. Y chromosome genes are highlighted in green.


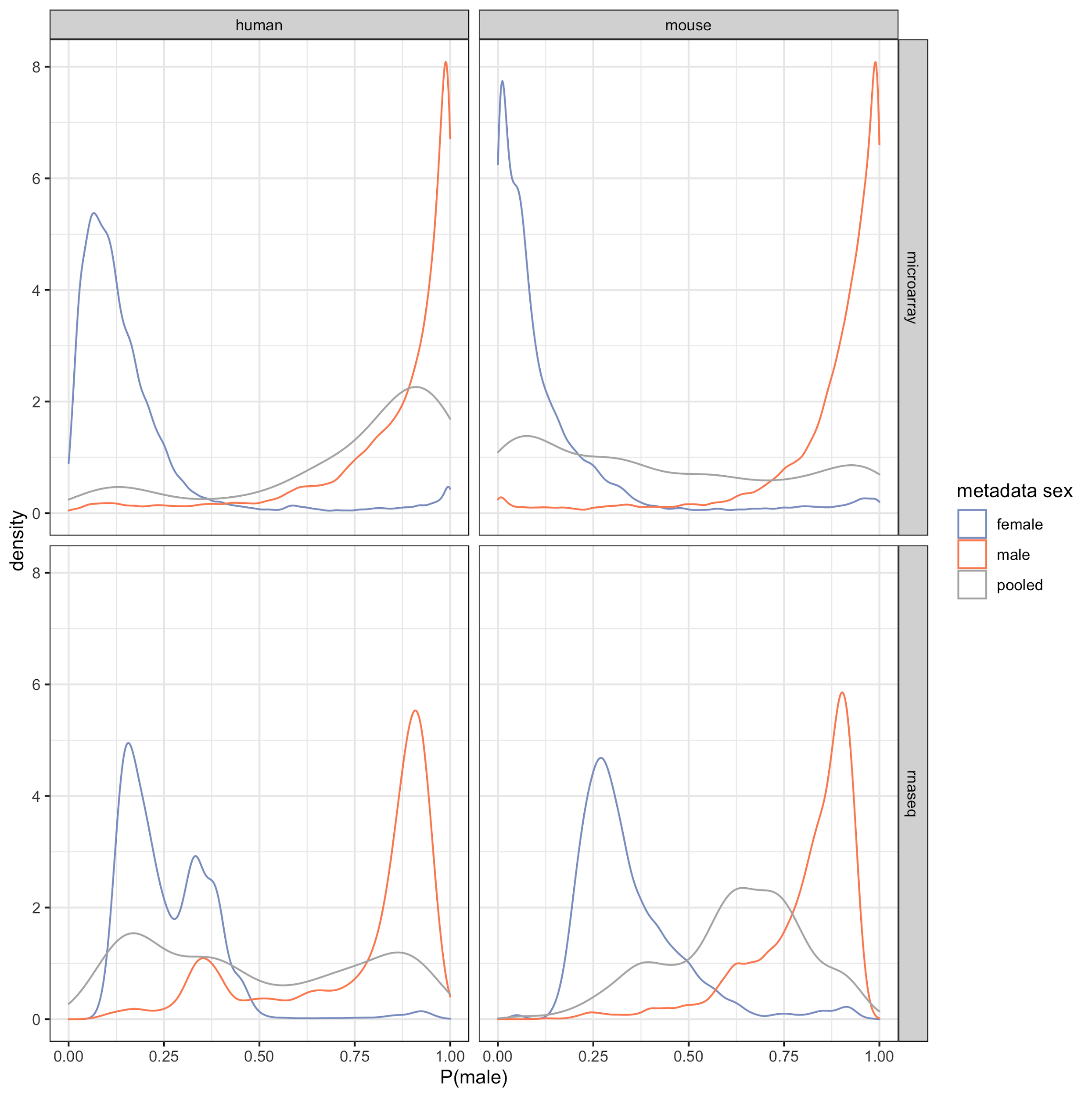


**Supplementary Figure 7.** Sample sex score distributions of female, male, and pooled samples by organism and technology type.


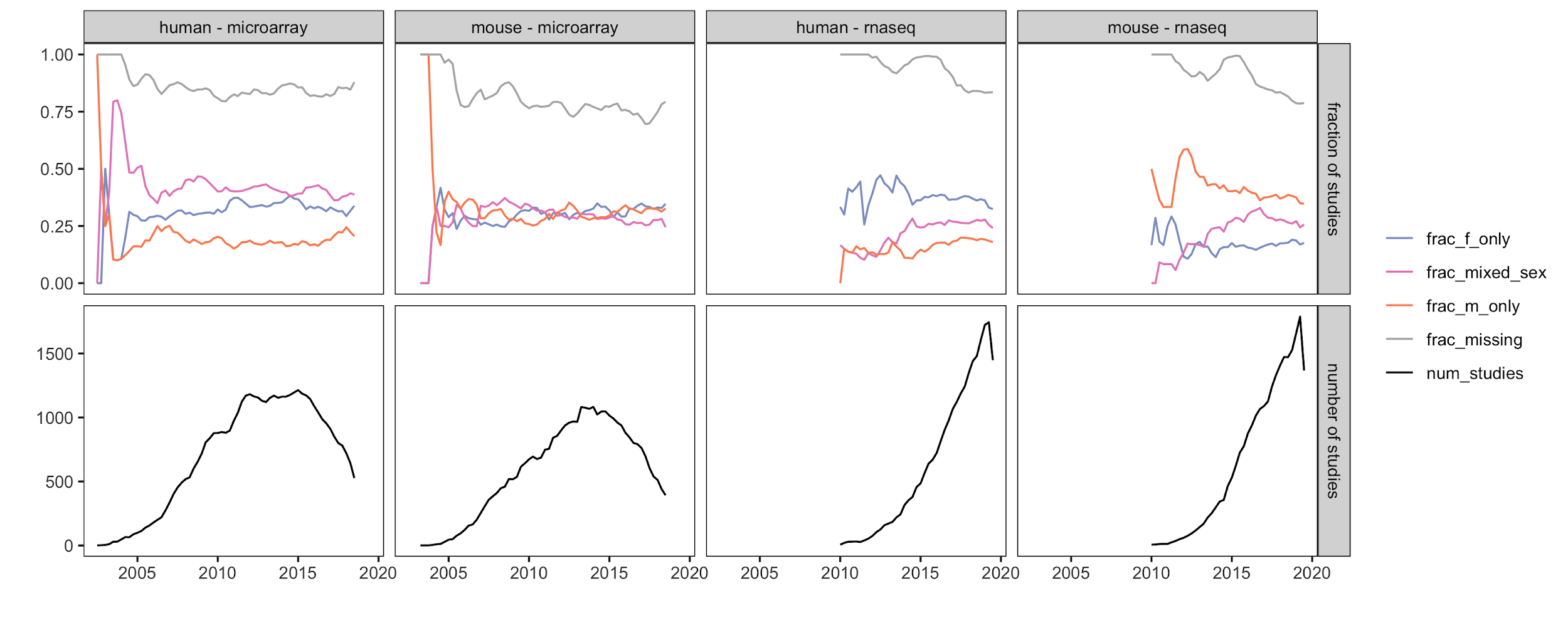


**Supplementary Figure 8.** Sex breakdown over time, by organism and data type. The top panel shows the fraction of studies with either missing sex labels (gray), or imputed female-only (blue), male-only (orange), or mixed sex (pink) samples. The bottom panel provides the total number of studies over time. Data were smoothed using a three-month window.


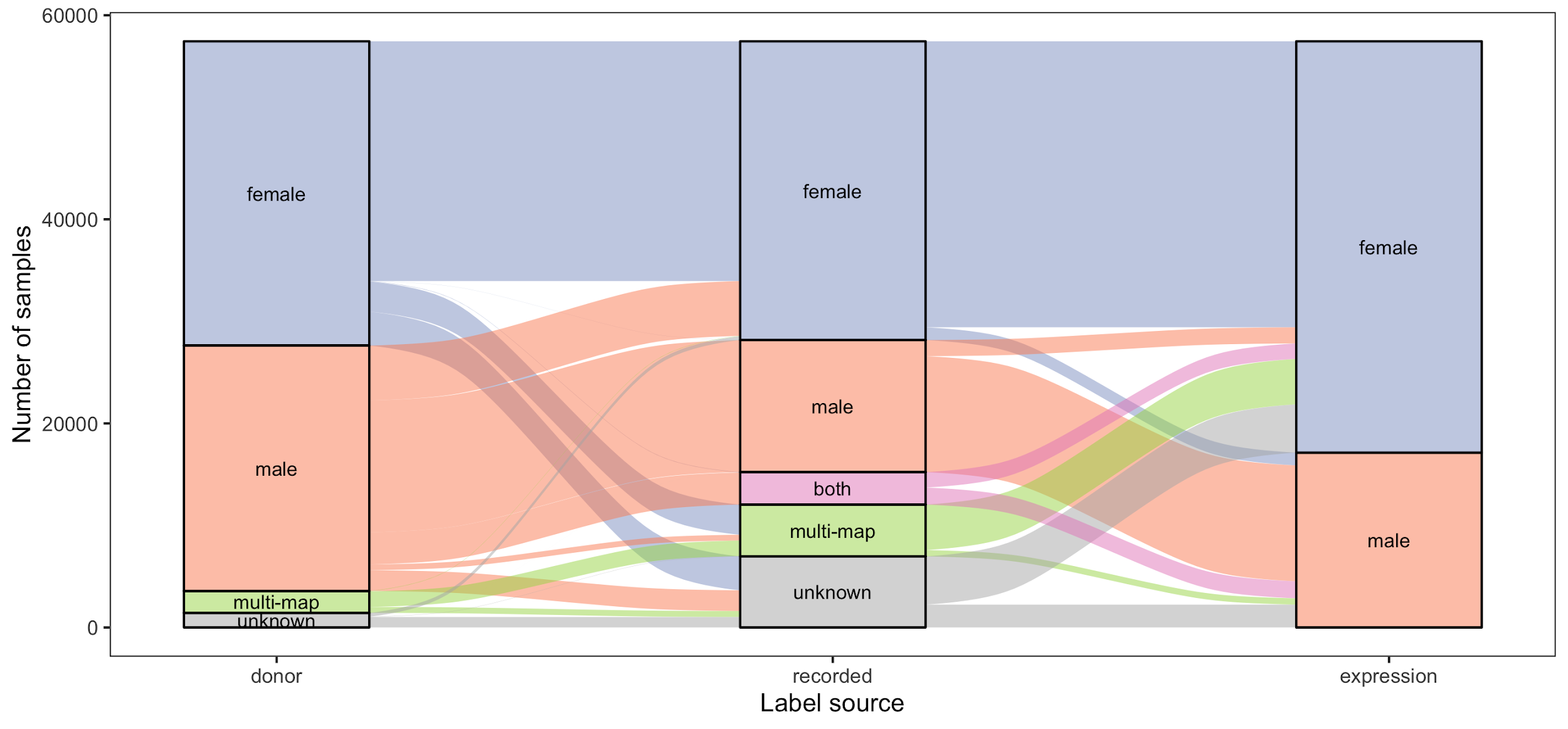


**Supplementary Figure 9.** Alluvial diagram showing cell line sex label “switching” and comparison of expression imputed sex labels to annotations of donor and recorded sex for that cell line.

A)
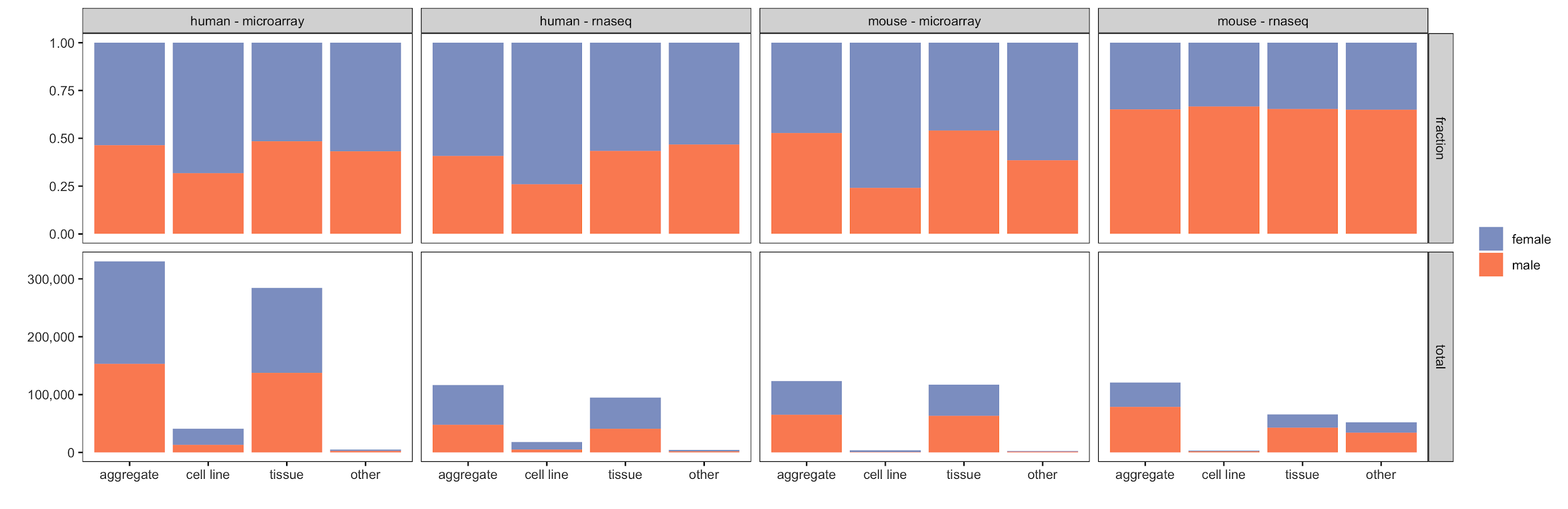


B)


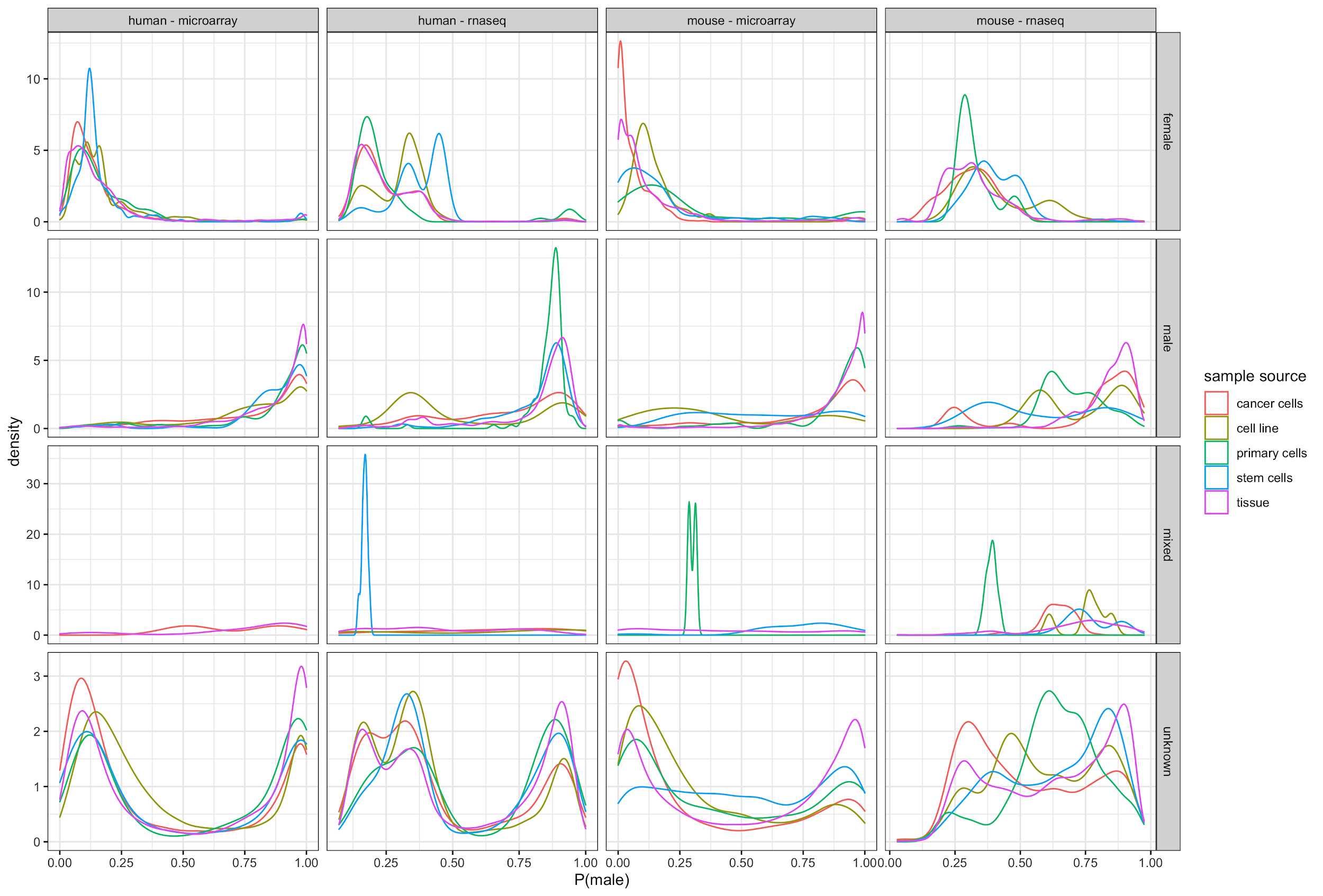


**Supplementary Figure 10.** A) Sex breakdown overall and in cell line versus tissue data. Bars are colored female (dark blue) or male (orange) to indicate the fraction (top row) or count (bottom row) of samples of that sex. B) Distribution of sample sex scores in cancer (red), cell line (yellow), primary cell (green), stem cell (blue), and tissue (purple) samples; separated by metadata sex (rows), and organism/data source (columns).

A)
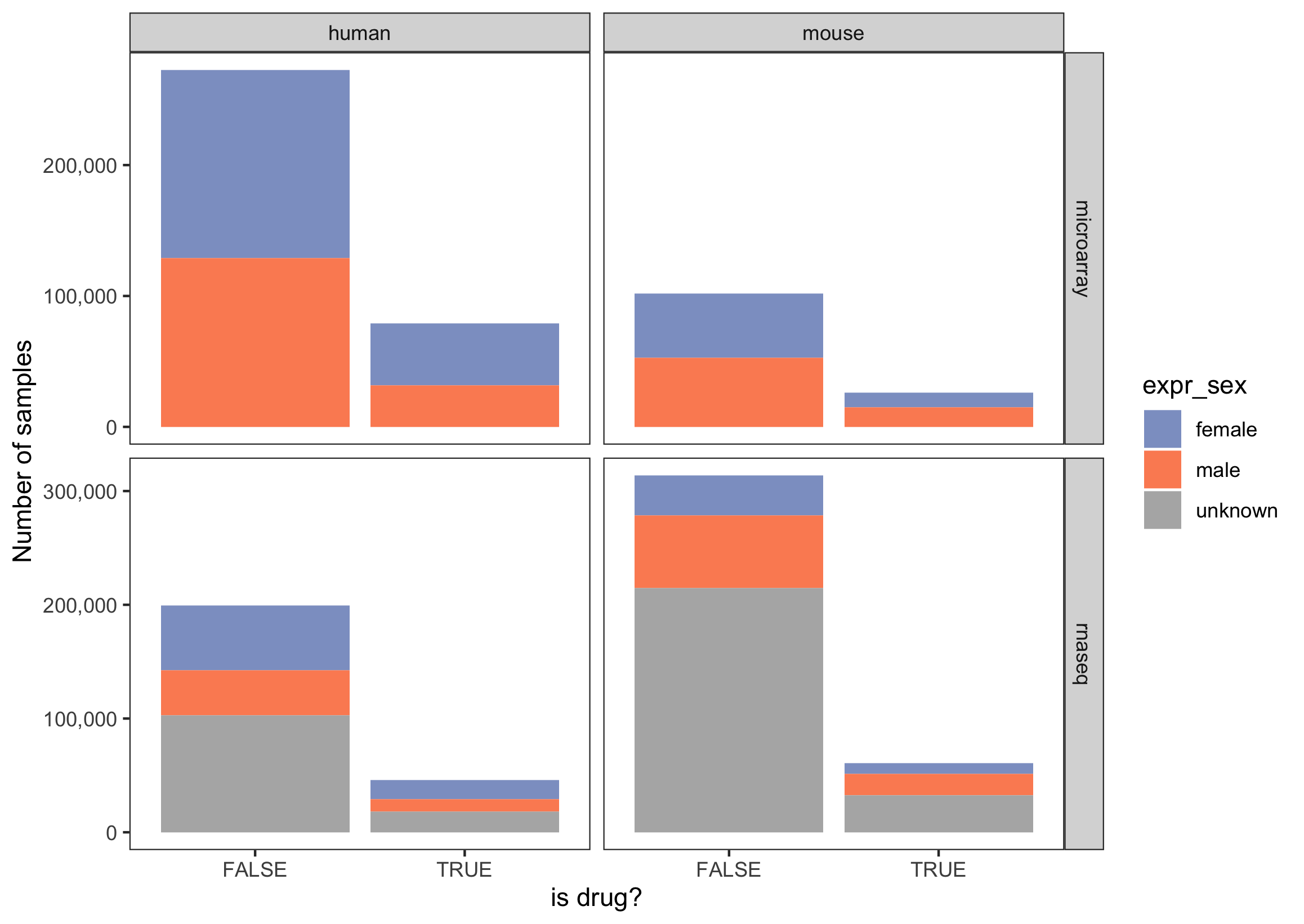


B)
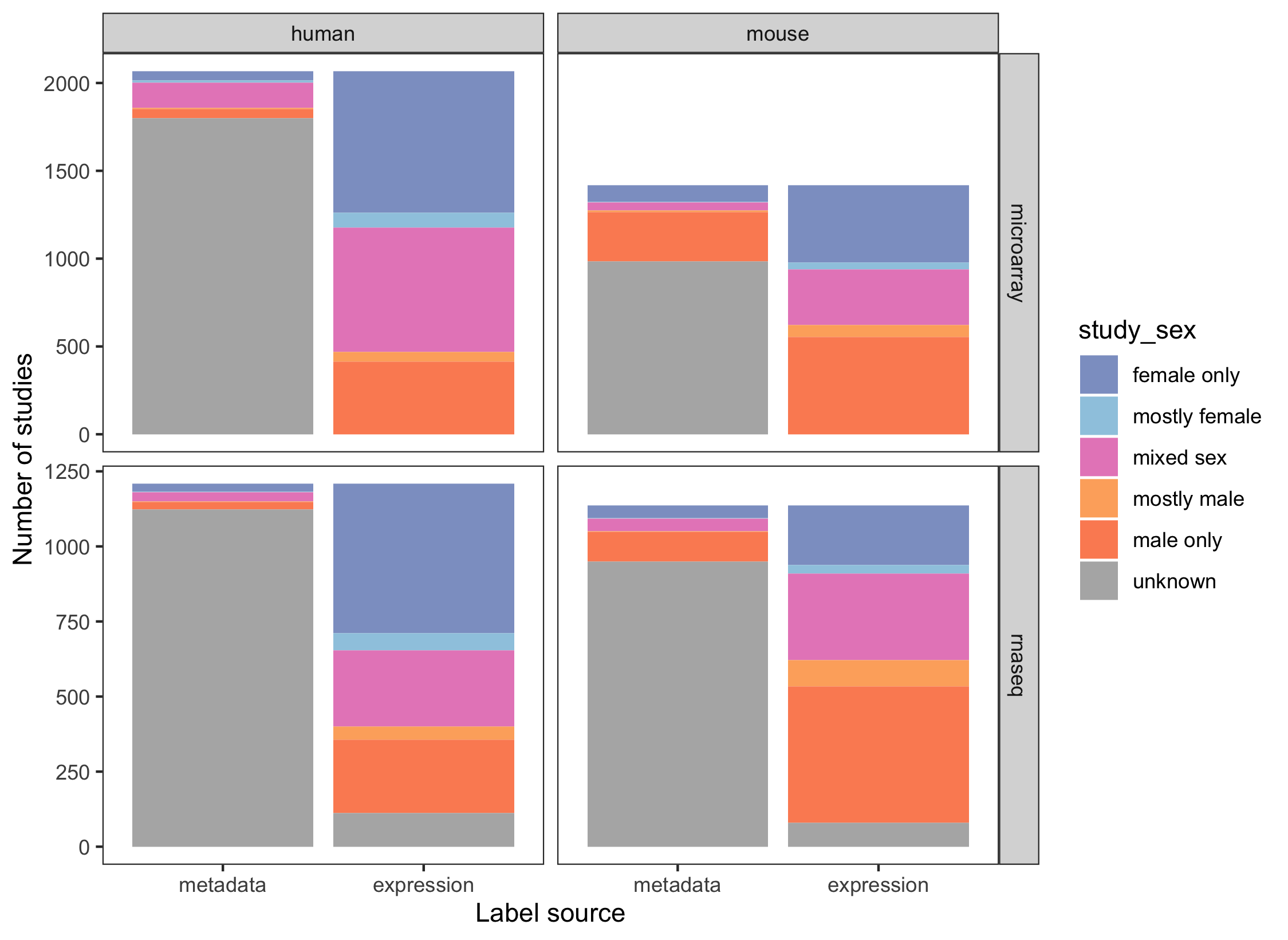


**Supplementary Figure 11.** Sex breakdown of drug samples (A) and studies (B) for *drug mention* data (see Methods 5-1).

A)
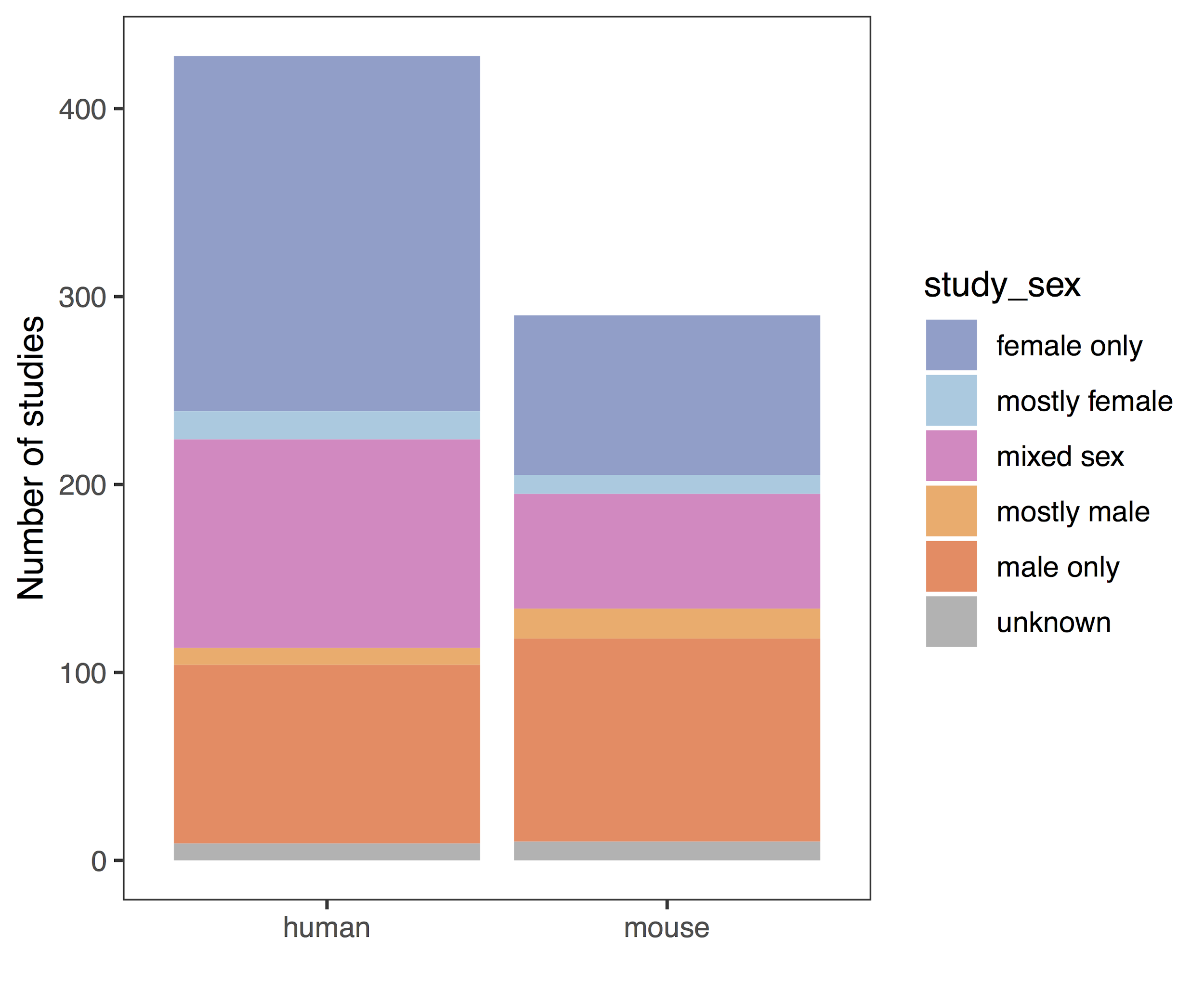
B)
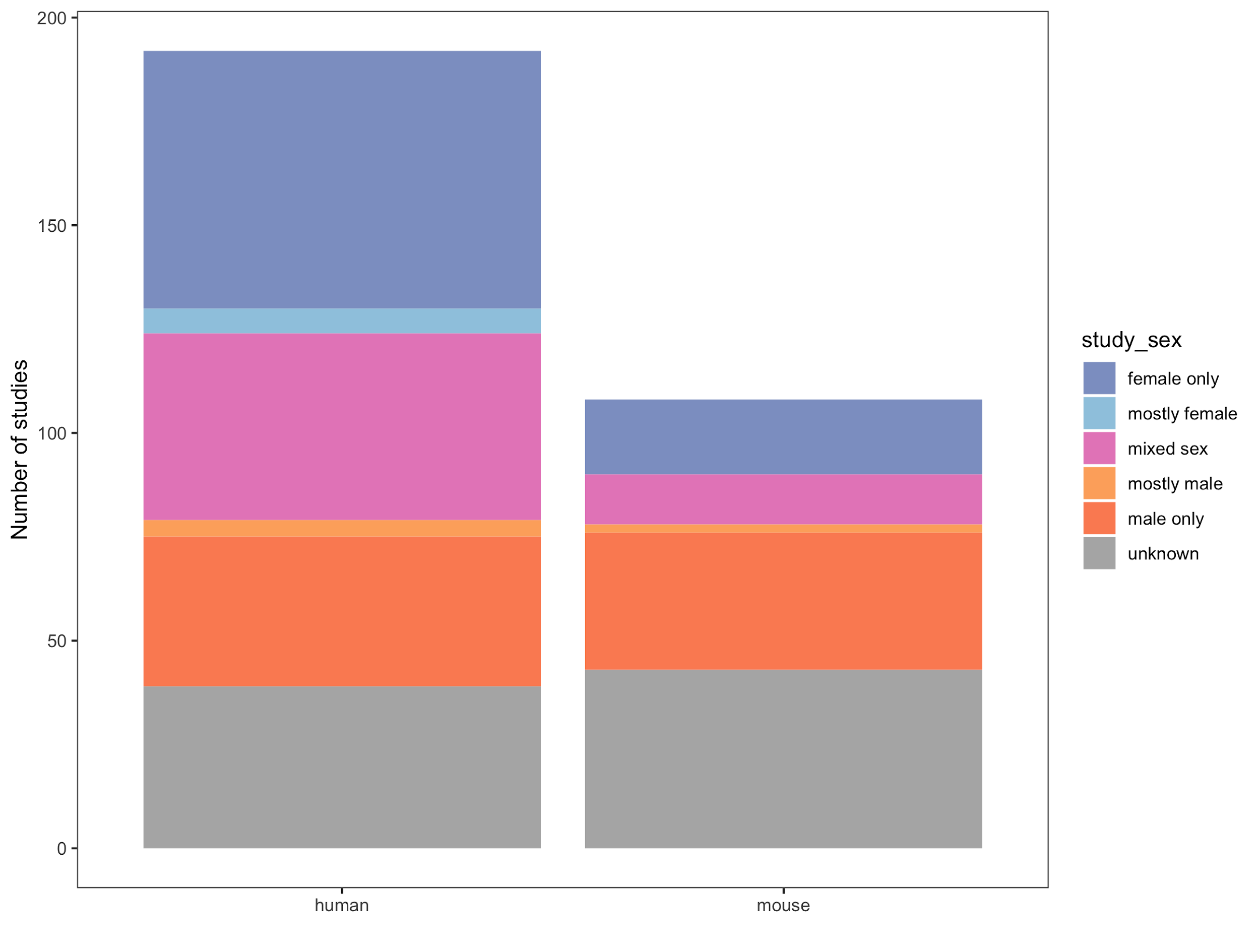


C)
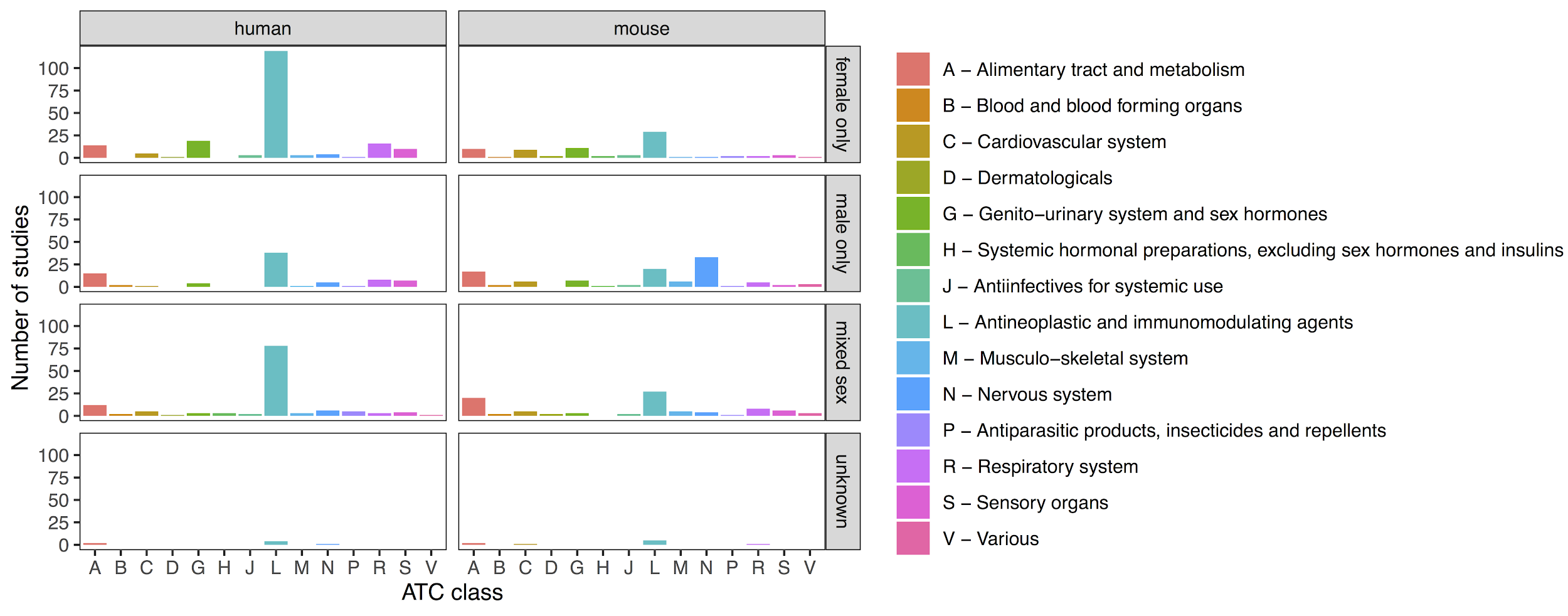


D)
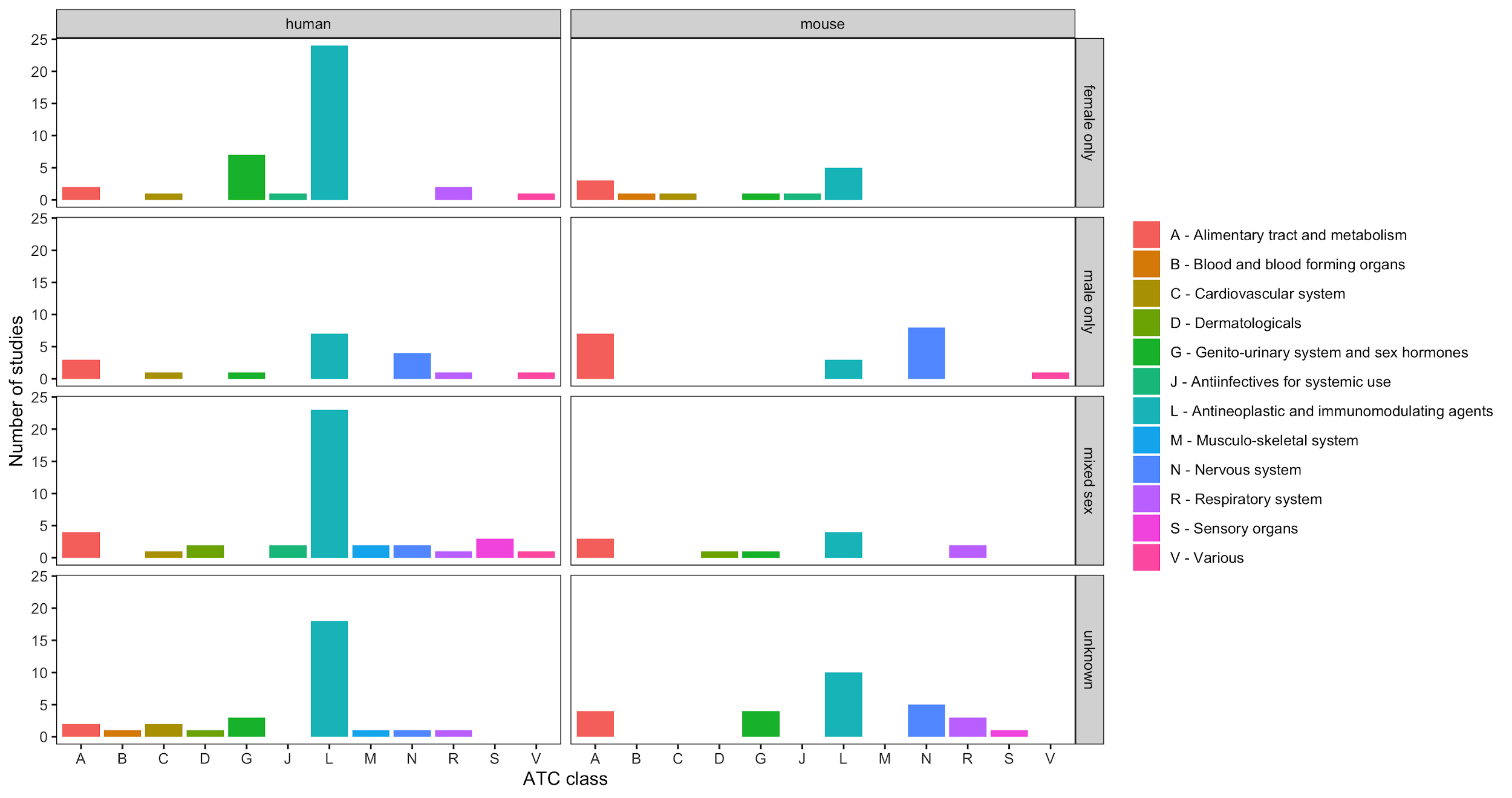


**Supplementary Figure 12.** Sex breakdown of samples directly mapping to drugs (*drug exposure studies* - see Methods 5-1) and manually curated drug data from Wang et al. [(Wang et al. 2016)](https://paperpile.com/c/F5x4Ty/wK0t) matches patterns seen in drug breakdown data in terms of overall study sex breakdown (A,B) and ATC class breakdown (C, D for drug exposure studies and Wang et al respectively).

A)
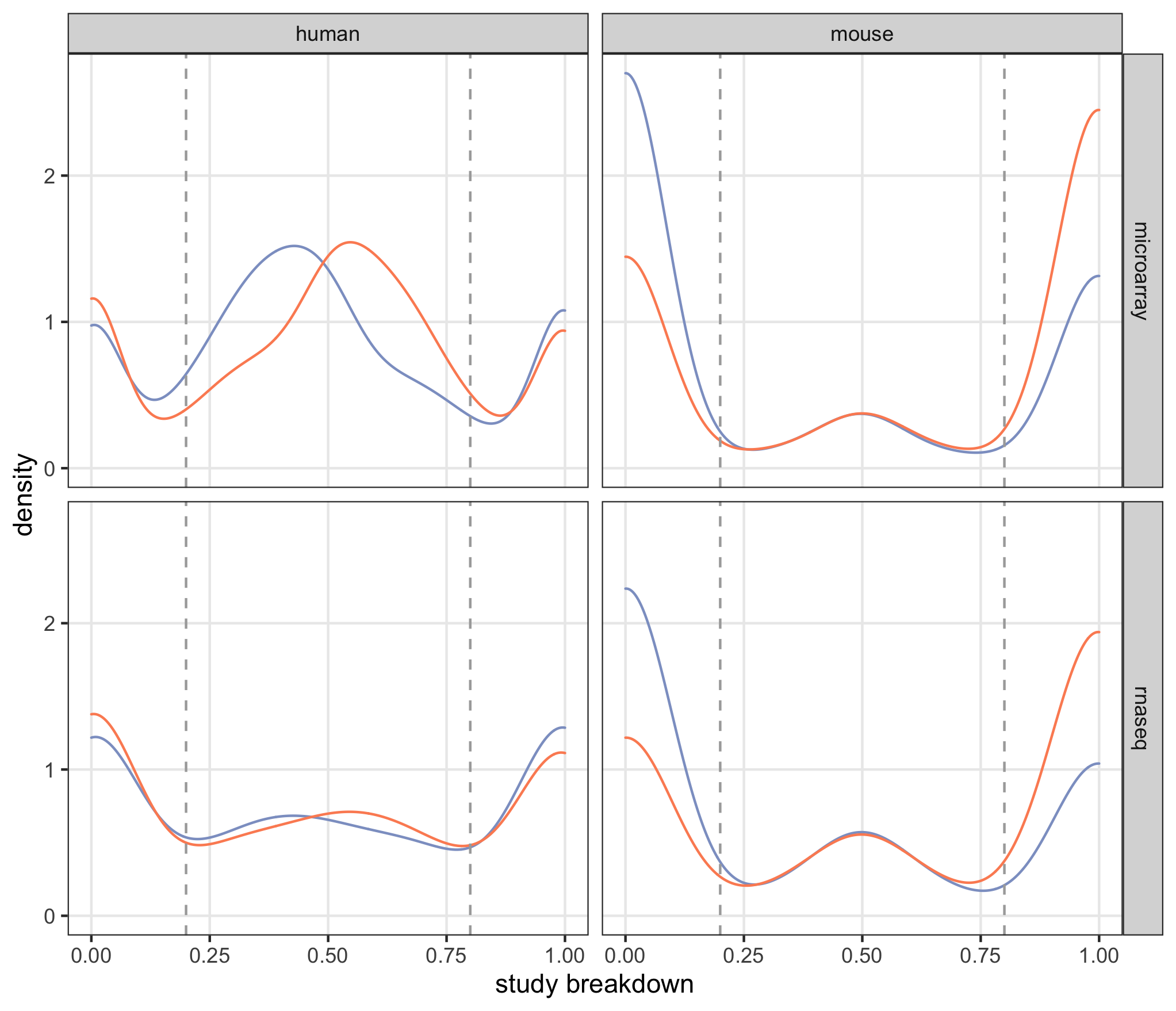
 B)
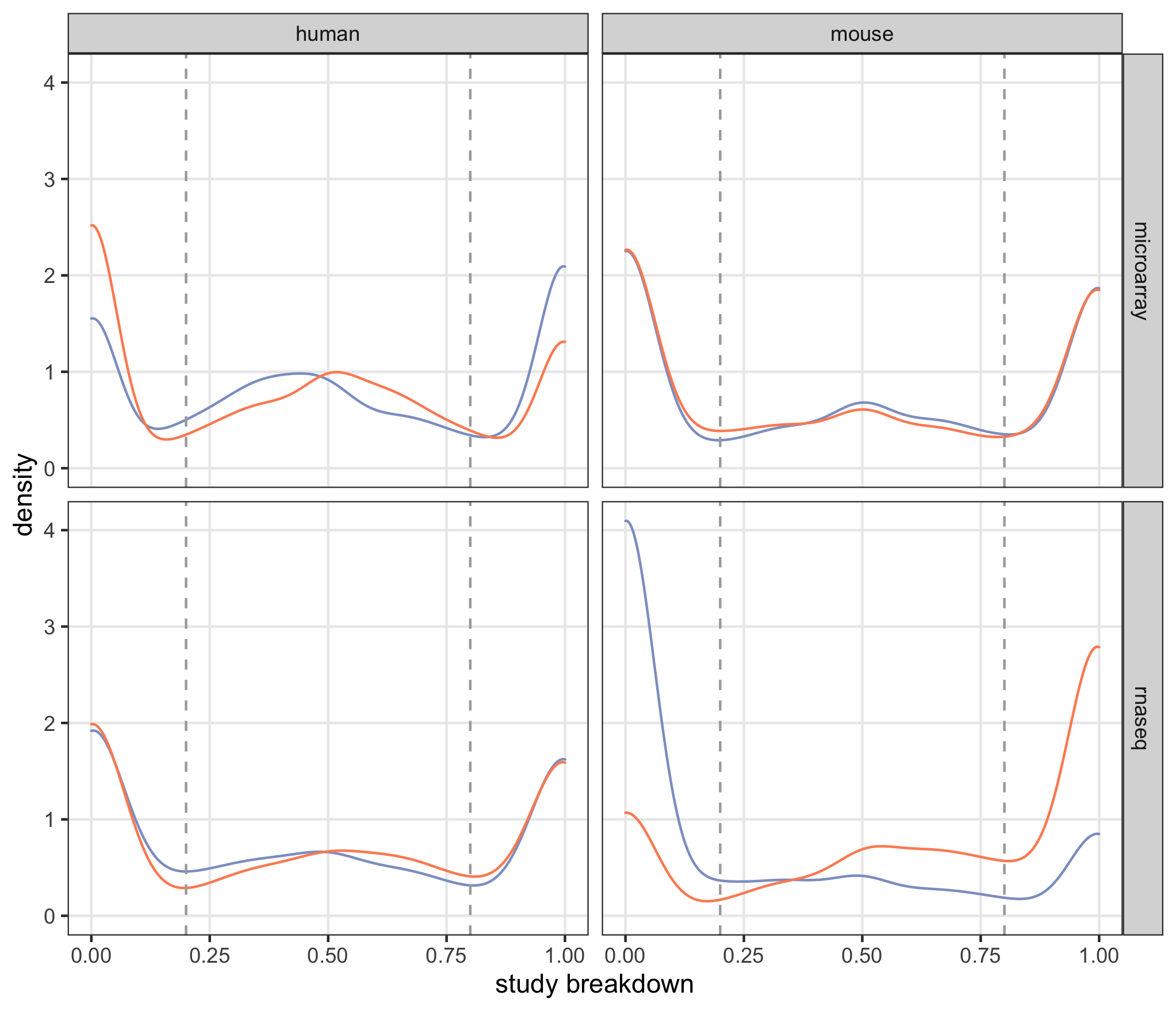

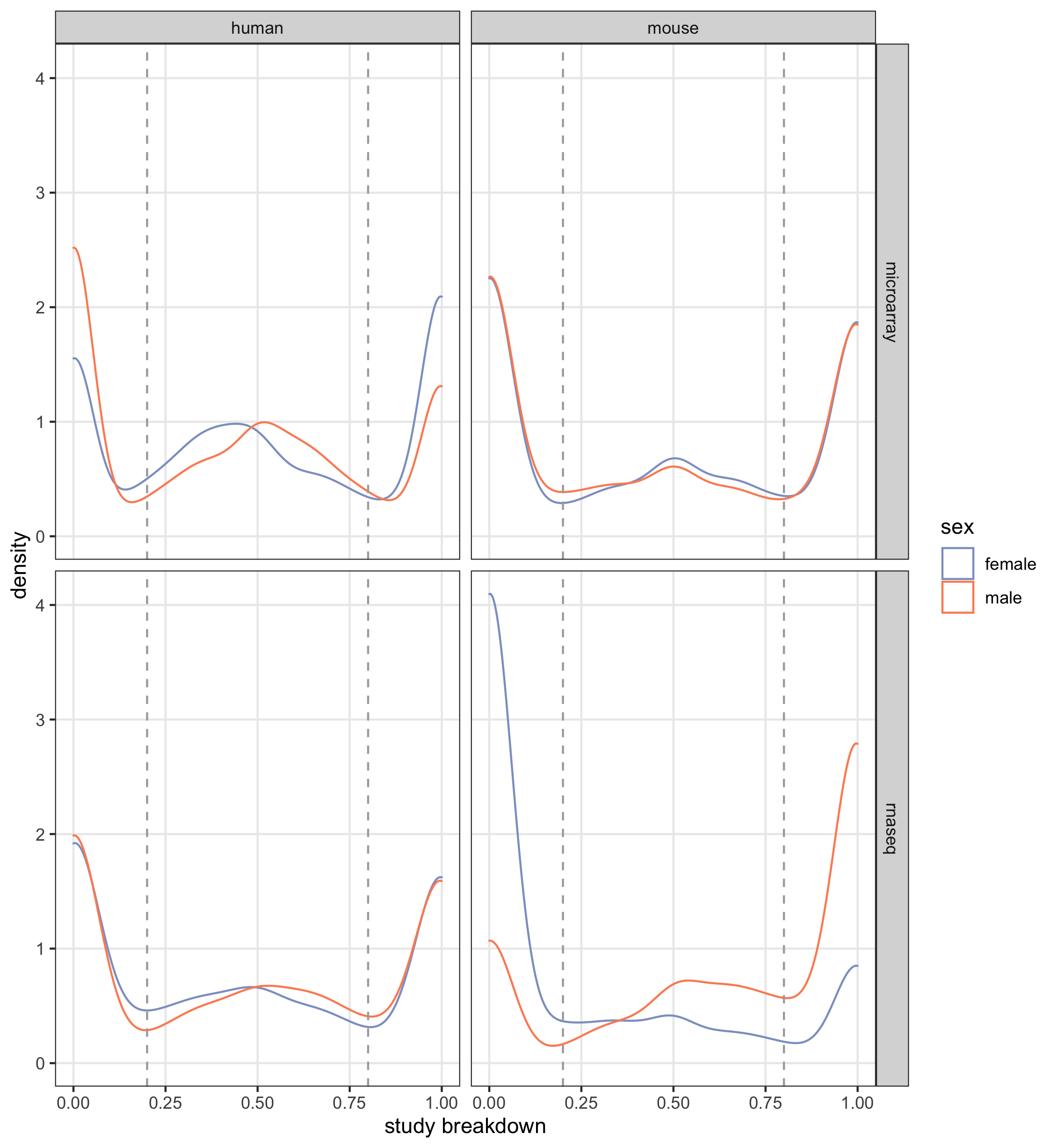


**Supplementary Figure 13.** Distributions of study sex breakdown For studies that are not designated as “unlabeled”, we show the proportions female (purple) and male (orange) based on metadata (A) and expression (B) labels, We include dotted gray lines at 0.2 and 0.8 to show the cutoffs we selected for identifying studies that are mostly- male or mostly- female. Data from human and mouse and microarray and RNA-seq are plotted separately.

A)
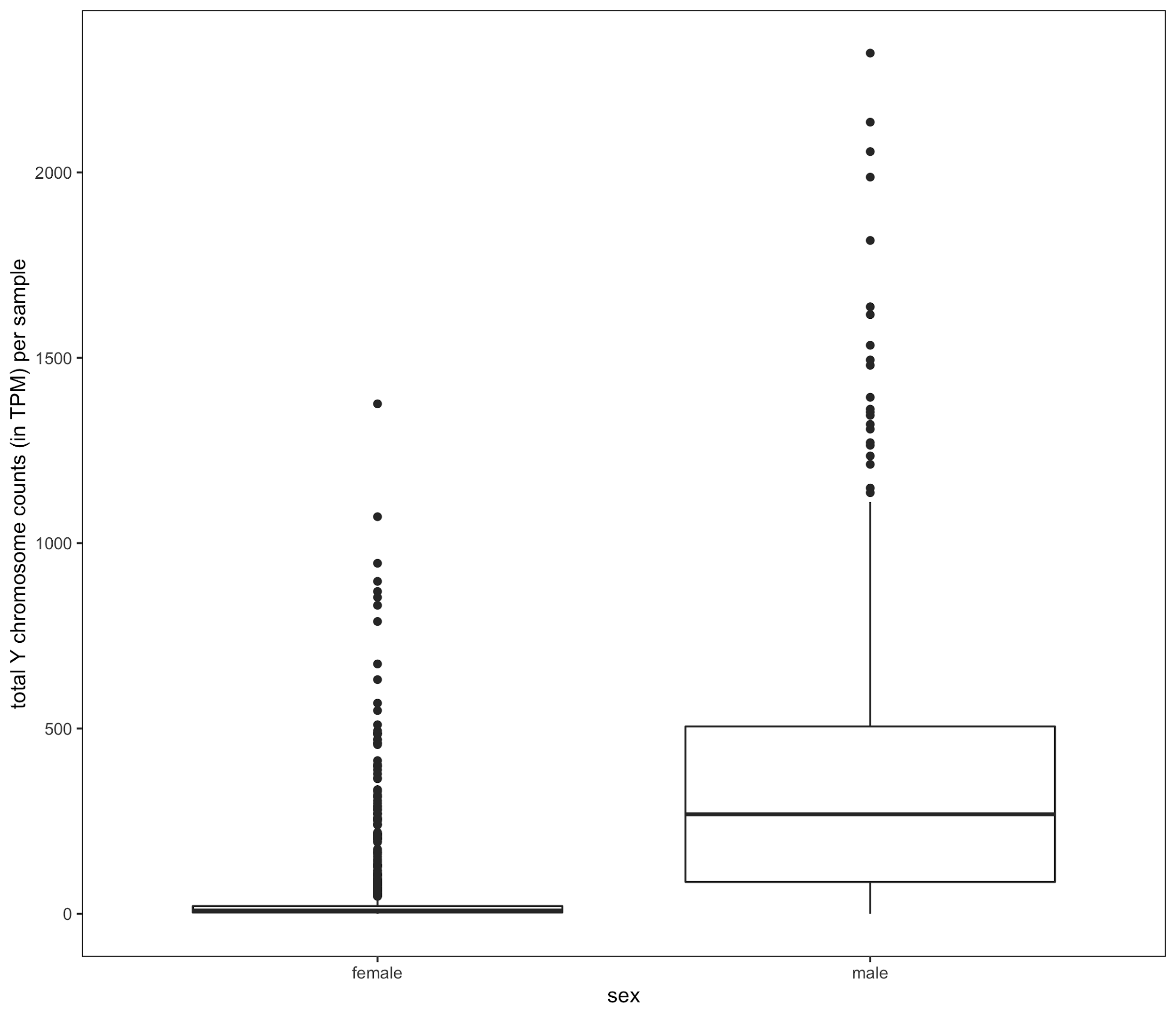
 B)
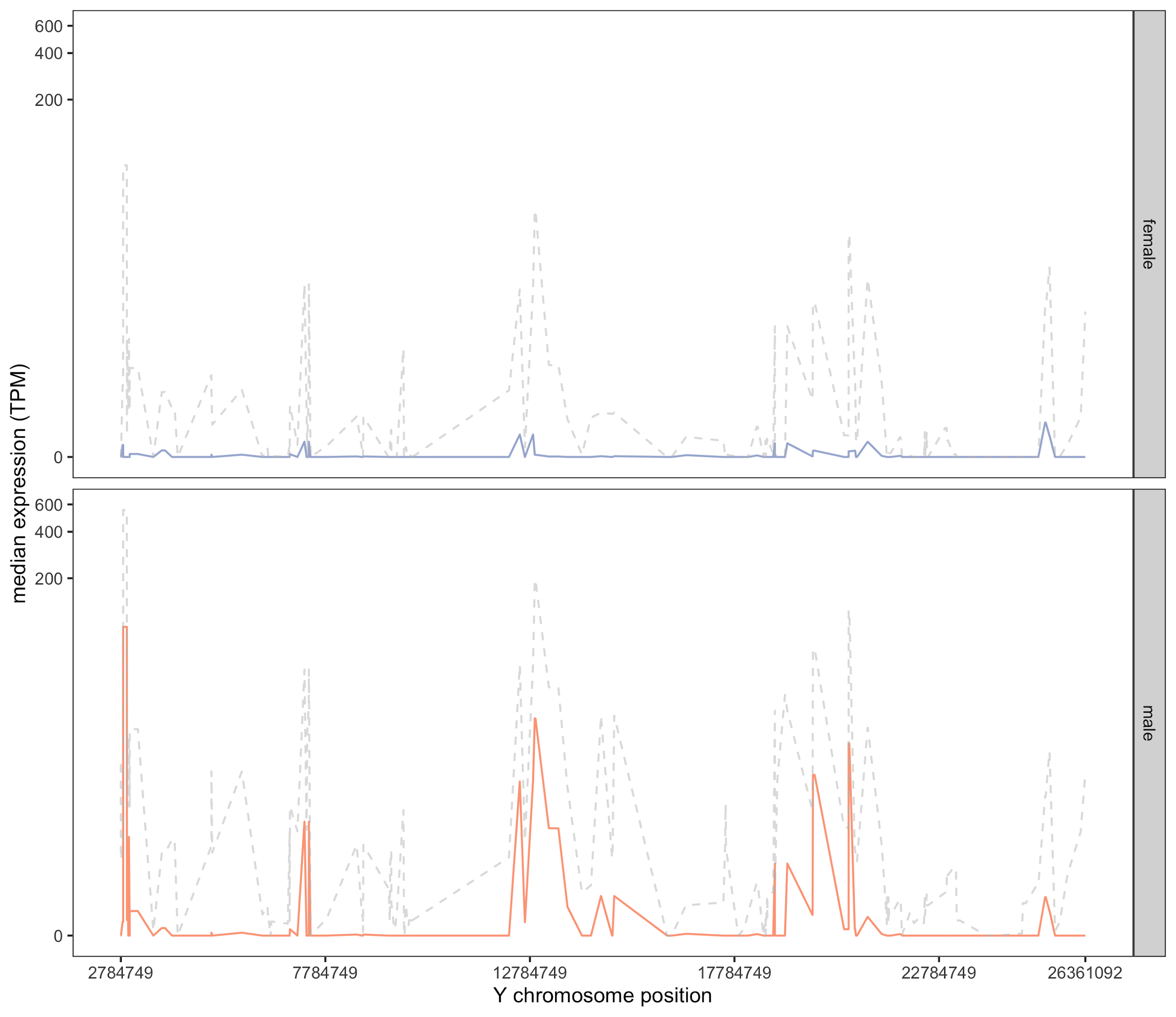


**Supplementary Figure 14.** Distribution of Y chromosome read counts in males and females in human training data. A) Overall counts per sample (in TPM). B) Base pair location of counts across the Y chromosome. For males (orange) and females (purple) separately, the median counts (in TPM) across the training data is shown with a solid line and the 95 percentile across samples is shown as a dotted gray line.


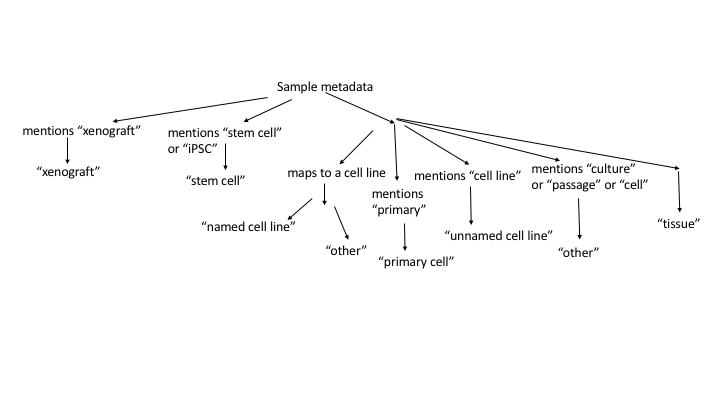


**Supplementary Figure 15.** Sample source assignment. Diagram showing logic of how samples were assigned to a sample source type based on metadata.
